# Simultaneous online monitoring of viscosity and oxygen transfer rate in shake flask cultures

**DOI:** 10.1186/s13036-025-00552-6

**Published:** 2025-08-22

**Authors:** René Hanke, Michaela Sieben, Maurice Finger, Kilian Schnoor, Simon Jeßberger, Julia Weyand, Lluìs Coloma de la Fuente, Marcel Mann, Amizon Azizan, Udo Kosfeld, Jochen Büchs

**Affiliations:** 1 AVT.BioVT - Chair of Biochemical Engineering, Rheinisch-Westfälische Technische Hochschule (RWTH) Aachen University, Aachen, Germany; 2 Faculty of Chemical Engineering, Universiti Teknologi MARA, Shah Alam, 40450 Selangor Malaysia

**Keywords:** Bioprocess, Optical measurement, *Paenibacillus polymyxa*, Process analytical technology (PAT), Respiratory activity monitoring system (RAMOS), Rheology, *Trichoderma reesei*, Viscosity monitoring online system (ViMOS), *Xanthomonas campestris*

## Abstract

Shake flasks are among the most relevant culture vessels for early-stage process development of viscous microbial cultures. While online process monitoring systems are available for temperature, pH, biomass concentration, dissolved oxygen tension and respiration activity, online measuring techniques for viscosity are not yet commercially available. Especially during the production of biopolymers and the cultivation of filamentous fungi or bacteria, quantification of fermentation broth viscosity is essential to ensure adequate mixing as well as gas/liquid mass and heat transfer. In this work, a previously developed quantitative online viscosity measurement technique, termed ViMOS, is refined to monitor the apparent viscosity of up to eight shake flask cultures in parallel. In addition, the necessary preparation to ensure reproducible measurements is elucidated. By cultivating the two exopolysaccharide forming bacterial strains, *Paenibacillus polymyxa* and *Xanthomonas campestris*, as well as the filamentous fungus *Trichoderma reesei*, the ViMOS was successfully validated for viscosity values up to 120 mPa·s. The combination with oxygen transfer rate monitoring via a RAMOS device allowed to detect microbial growth phases, oxygen limitations, biopolymer production and degradation, as well as the morphological development of filamentous cultures. This dual online monitoring has the potential to improve screening conditions and simplify scale-up procedures of small-scale bioprocesses.

**Supplementary Information:**

The online version contains supplementary material available at 10.1186/s13036-025-00552-6.

## Background

### Online monitoring of viscosity in shake flasks

Being easy to handle, inexpensive and well-characterized [1, 2, 3, 4, 5, 6], shake flasks are standard cultivation vessels used to investigate microbial culture behavior in a small scale. Common procedures in microbial bioprocess development include microbial strain screening, culture media optimization, process parameter selection and generation of pre- or starter cultures for stirred tank fermentations [7]. For this purpose, online methods have been developed to measure key parameters like oxygen availability [8, 9, 10], biomass concentration [11, 12], and pH [13] in shake flasks, in order to accelerate and improve the bioprocess development [13].

The viscosity of the culture broth is a crucial process parameter in microbial cultivations for biopolymer production, in processes involving microorganisms with a filamentous growth morphology and in fermentations with fibrous as well as polymeric substrates [14, 15, 16, 17, 18, 19]. Commercially relevant examples of biopolymers are xanthan secreted by *Xanthomonas campestris* [20], alginate produced by *Azotobacter vinelandii* [21, 22] or poly-γ-glutamic acid (γ-PGA) synthesized by several *Bacillus* strains [17, 18, 23, 24]. Furthermore, studies of plant suspension cultures have reported that high cell concentration or the secretion of polymers enhances the viscosity [25, 26]. When cultivating bacteria and fungi with filamentous morphology, the interaction between mycelial growth and morphological development influences the viscosity of the culture broth [16, 27, 28, 29, 30]. These changes play a role e.g. during the production of antibiotics using *Penicillium chrysogenum* [31, 32], citric acid using *Aspergillus niger* [33, 34] or enzyme mixes for biofuel synthesis using *Trichoderma reesei* [35, 36, 37]. Simultaneous filamentous growth and polymer formation reveal an even more complex viscosity development during bioprocesses. The fungi *Aureobasidium pullulans* and *Schizophyllum commune*, while possessing a filamentous morphology, also produce the polymers pullulan and schizophyllan, respectively [38, 39].

An increasing broth viscosity in shake flask cultures strongly influences the fluid flow, the thickness of the liquid film on the hydrophilic glass wall and mixing performance [14, 40, 41, 42] as well as gas/liquid mass and heat transfer [43, 44, 45, 46]. Elevated viscosity can consequently lead to a lack of oxygen and nutrient supply, causing metabolic changes in the organisms and reducing both cell growth and productivity [31, 34, 46]. Therefore, an understanding of the culture rheology, or at least knowledge of apparent viscosity, is an important prerequisite to develop an appropriate process design that helps to overcome the challenges of viscous fermentations [26, 31, 47].

While online monitoring of viscosity in stirred tank reactors is realized via process viscometry, viscosity probes [48, 49] or calculated from heat transfer capacity [50], these methods have not been established for shake flasks yet. Therefore, most laboratories must rely on taking samples during several time points of cultivation and perform offline measurements using a conventional rheometer. Unfortunately, frequent sampling often requires interrupting the shaking process, risking detrimental effects on the biological culture, such as oxygen limitation or broth acidification [45, 51]. In addition, rheological offline measurements have to be performed quite fast, to avoid rheological changes of the sample [27, 52, 53]. For shake flask cultivations, Lotter and Büchs [54] developed an online viscosity measurement technique based on non-invasive power input measurements [15, 24]. However, this method cannot be combined with other online monitoring techniques, it does not allow taking samples during the measurements and requires 9 to 14 shake flasks to be operated in parallel and under identical conditions. More importantly, the measurement of viscosity based on the power input requires a minimum mass for an accurate measurement. During the last years, optical methods for online viscosity measurements in shake flasks have been developed, based on the detection of the leading edge of the bulk liquid relative to the direction of the centrifugal force [55, 56, 57]. While the concept by Ladner et al. (2019) [56] may be combined with online pH, DOT, and/or OTR monitoring, it requires the addition of oxygen-sensitive nanoparticles to the biological cultivation. In contrast, the method demonstrated by Sieben et al. (2019) [55] relies on the absorption of near-infrared light at 970 nm by the aqueous culture broth. It may serve as a less expensive method compared to Ladner et al. (2019), as it does not require the use of oxygen‐sensitive nanoparticles [55, 56]. Recently, Dinter et al. (2024) presented a more advanced method of viscosity monitoring based on the leading edge angle of the bulk liquid without the need for fluorescent nanoparticles [57]. All these optical methods share the major advantage compared to the power input measurement by Lotter et al. (2004) that each shake flask serves as individual cultivation vessel with a unique filling volume and content [54, 55, 56, 57]. An overview of the discussed methods for online viscosity measurement in shake flasks is given in Supplementary Table S1. First results of the combined online monitoring of biological culture viscosity and oxygen transfer rate have already been demonstrated to guide media optimization and support metabolic engineering strategies for γ-PGA production using *Bacillus subtilis* [17, 58]. Analogously to the commercially available RAMOS [9], the “Viscosity Monitoring Online System” developed by Sieben et al. (2019) is referred to as ViMOS [58]. While Halmschlag et al. (2020) [18] did not provide any validation of the shown viscosity values by traditional rheometer measurements, Hoffmann et al. (2022) [58] presented offline viscosity values that only showed the same qualitative course as the ViMOS values. Offline viscosity values were up to 50 % lower compared to online viscosity and a calibration of the ViMOS was mentioned but not further specified.

In this study, the combined online monitoring of biological culture viscosity and oxygen transfer rate, using the ViMOS and the RAMOS technology, is demonstrated for three viscous microbial model systems. First, the essential preparation steps to ensure reliable measurement of apparent viscosity are outlined, including shake flask orientation and pretreatment. Second, the calibration function is adjusted to cover a viscosity range from 0.9 to 200.6 mPa·s. Third, the application of the ViMOS and RAMOS combination for bacterial biopolymer production and cultivation of filamentous fungi is demonstrated. While previous studies applied the ViMOS for bacterial cultivations up to only 42 mPa∙s [17, 18, 58], this study investigates bacterial cultivations up to 150 mPa∙s, and for the first time, demonstrates an online viscosity monitoring in shake flasks for cultures of filamentous fungi.

### Liquid distribution in orbitally shaken flasks depends on liquid viscosity

**Fig. 1:**
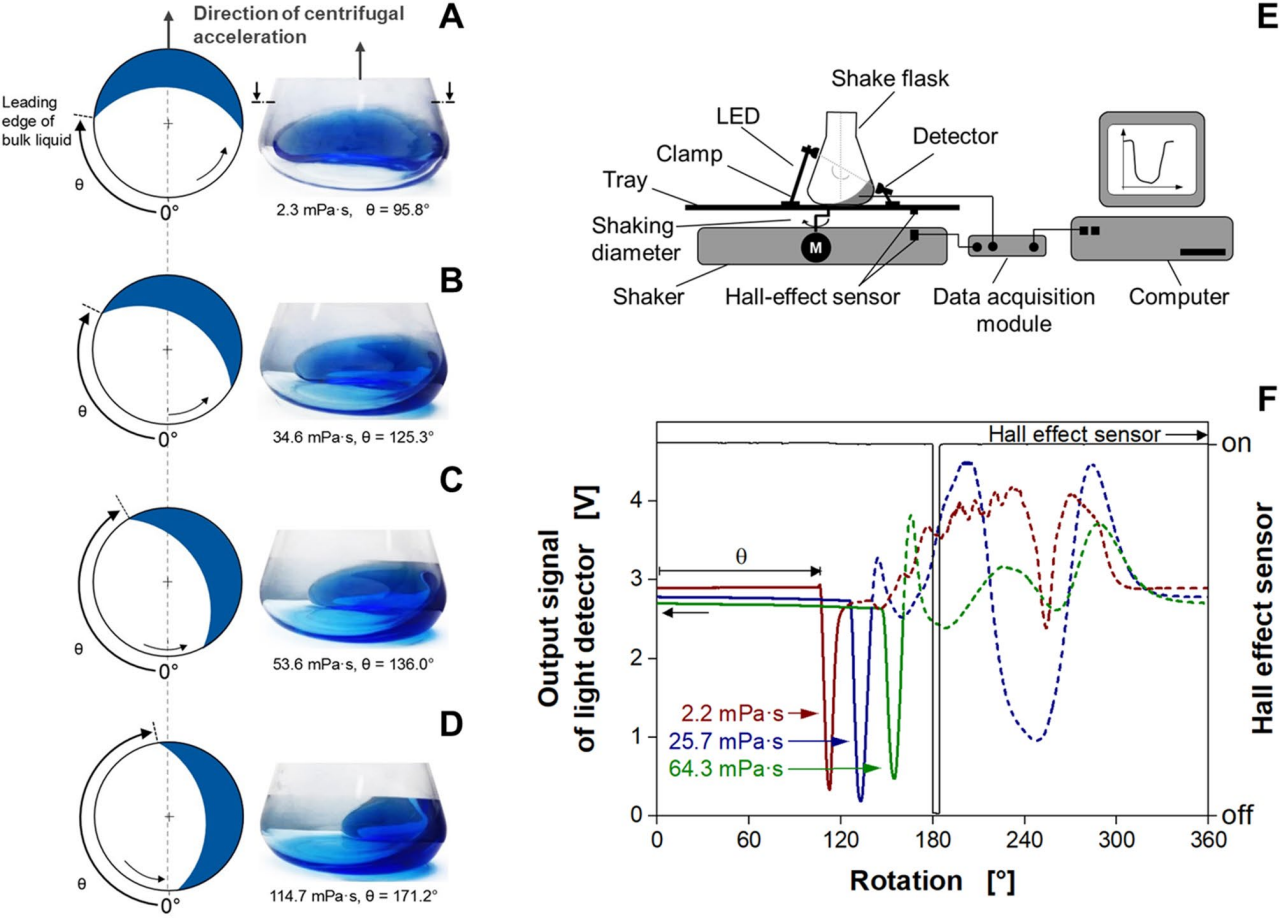
Operating principle of the online viscosity measurement device ViMOS. (**A–D**) Bulk liquid distribution in a rotating shake flask at different viscosities. Polyvinylpyrrolidone (PVP) solutions of four concentrations, *V*_*K*_ = 250 mL, *V*_*L*_ = 30 mL, *n* = 150 rpm, *d*_*0*_ = 50 mm, *T* = 25 °C. Schematic drawings show a top view of a shake flask with a rotating bulk liquid. *θ* indicates the angle between the zero point and the leading edge of the rotating bulk liquid. The shake flask was placed in an orbital shaker with a counterclockwise rotational path. Photographs were taken in the direction of the centrifugal acceleration, using a rotating camera (Tan et al., 2011; Seletzky et al., 2006) [40, 62]. The angle *θ* and corresponding viscosity are indicated below each flask. (**E**) Schematic illustration of the transmitted light measuring setup. Light source and detector are positioned on opposite sides of the shake flask. (**F**) Raw signals of the measured angle of the leading edge of the bulk liquid in shake flasks, for PVP solutions with different concentrations. *V*_*F*_ = 250 mL, *V*_*L*_ = 20 mL, *n* = 300 min^− 1^, *d*_*0*_ = 50 mm, *T* = 30 °C. Parts of this figure have been adapted from Sieben et al. (2019) [55]

Shake flasks are orbitally agitated in biological cultivation processes to ensure sufficient mixing and, hence, oxygen and nutrient supply. The orbital movement of the shake flask induces a rotation of the bulk liquid due to its inertia (Fig. 1A-D). A thin liquid film is formed on the inner hydrophilic glass wall [5, 14, 59]. At water-like viscosities, the center of mass of the bulk liquid is oriented along the direction of the centrifugal force (Fig. 1A). With increasing viscosity, the bulk liquid rotates shifted (delayed) with respect to the direction of centrifugal acceleration (Fig. 1B-D). Additionally, the liquid film adhering on the hydrophilic wall of the shake flask increases in thickness [14], while a part of the bulk liquid remains on the bottom of the sample vessel (Fig. 1B-D). Further increasing viscosities can result in a collapse of the liquid movement, if the liquid system turns “out-of-phase” [2]. The out-of-phase phenomenon leads to unfavorable operating conditions, since the specific power consumption as well as gas/liquid mass transfer are dramatically reduced. During “out-of-phase” conditions, an online viscosity measurement is not possible. The relationship between the leading edge angle of the bulk liquid and the liquid film thickness for different viscosities and the transition to “out-of-phase” conditions has been thoroughly characterized during the last years [14, 41, 42, 55, 56, 57, 60, 61]. To calculate the onset of unfavorable conditions, Büchs et al. (2000) [2, 3] introduced the so-called phase number *Ph* (Eq. 1).

1 $$\:Ph=\frac{{d}_{0}}{d}\left(1+3{\text{log}}_{10}\left[\frac{\rho\:n{d}^{2}}{{\eta\:}_{\text{a}\text{p}\text{p}}}\cdot\:\frac{\pi\:}{2}{\left(1-\sqrt{1-\frac{4}{\pi\:}{\left(\frac{{V}_{L}^{\frac{1}{3}}}{d}\right)}^{2}}\right)}^{2}\:\right]\right)$$

2 $$\:F{r}_{\text{a}}=\frac{{\left(2\pi\:n\right)}^{2}\cdot\:{d}_{0}}{2g}$$

Apart from the apparent viscosity *η*_*app*_, the phase number *Ph* is influenced by the maximal inner shake flask diameter *d*, shaking frequency *n*, shaking diameter *d*_*0*_ and liquid volume *V*_*L*_ as well as culture density *ρ*. According to Büchs et al. (2000), a shake flask culture is out-of-phase, when the phase number *Ph* falls below a value of 1.26, which is called the critical Phase number (*Ph*_*crit*_). The calculation of *Ph* is only valid for axial Froude numbers (*Fr*_*a*_) > 0.4 (Eq. 2). A more recent study by Azizan et al. (2019) propose a less conservative threshold Phase number *Ph*_*crit*_ of 0.91 [60]. If shaking conditions are known, the apparent viscosity value, above which “out-of-phase” conditions appear, is called critical viscosity (*η*_*crit*_).

### Detection of the liquid distribution in shake flasks via transmitted light measurement

Sieben et al. (2019) published a direct correlation between the position of the leading edge of the rotating bulk liquid and the viscosity of the liquid [55]. Since the leading edge of the bulk liquid has a distinct geometric shape, it can easily be detected and is taken as a reference point for the detection of the bulk liquid position. The angle between the zero point and the leading edge of the rotating bulk liquid is referred to as the leading edge angle *θ* (Fig. 1). To determine the position of the leading edge of the bulk liquid, a transmitted light measurement was chosen (Fig. 1E), as described in Sieben et al. (2019) [55]. A 250 mL Erlenmeyer flask without graduation was mounted on an orbital shaker (LS-X, Kühner AG, Birsfelden, Schweiz) equipped with a Hall effect sensor as a position marker and placed in a temperature-controlled hood. The light source and the detector were located on opposite sides of the shake flask, as shown in Fig. 1E. Any liquid passing in between the light source and the detector reduces the intensity of light that reaches the detector. The light source was a near-infrared LED (LD 274, *λ*_*max*_ = 950 nm, OSRAM Licht AG, München, Germany) to take advantage of the liquid water absorption band around 970 nm [63, 64, 65]. This approach is especially suitable for aqueous fermentation broths. The LED was mounted at a height of 70 mm (always being above the maximum liquid height), to avoid any interference of the rotating bulk liquid at this side of the shake flask. The corresponding infrared detector (203 FA, OSRAM Licht AG, München, Germany) was fixed at the opposite side at a height of 13 mm from the shake flask bottom. At this height, the 250 mL shake flask possesses its maximal radial diameter as the truncated cone merges into the quarter torus. The bulk liquid maximum extension inside the shake flask can also be found at this height. Therefore, this position is particularly suitable for measuring. From the infrared detector, the signal was forwarded together with the Hall effect sensor signal to a data acquisition module (NI USB 6210, National Instruments, Austin, TX, USA) that processed the data to a computer. A self-developed software based on LabVIEW™ (National Instruments, Austin, TX, USA) visualized and saved the data.

In a first “single shake flask” setup of this measuring device, angle meters were attached to both the LED and the detector, enabling the free adjustment of the angles with respect to the longitudinal axis of the shake flask (Supplementary Figure S1). A mathematical approach was used to identify preliminary orientations of LED and detector (Supplementary Figure S2). It was assumed that the highest signal intensity is achieved when the light emitted by the LED meets the plane of the detector perpendicularly. Since the distance between the detector and the LED as well as the height of the LED were known, the angles *β* and *γ* were calculated to be 53.5° and 126.5°, respectively. Subsequently, it was experimentally tested whether the light refraction on the shake flask glass and the liquid leads to another optimal alignment of LED and detector than the theoretical consideration. Using different shake flasks, filling volumes, shaking frequencies, and nine different fluids, 45 different combinations were tested with LED angles ranging from 123–131° and detector angles from 51–59° (Supplementary Figure S2, Supplementary Table S2 & S3). The experimental results reflected the theoretical consideration: the best signal was obtained at an angle pair of 53° (detector) and 126° (LED). Due to spatial restrictions, for an “eight shake flask” setup Supplementary Figure S3), only this fixed angle setting was used. Working with a multi-flask device offered not only the advantage of monitoring cultivations with identical operating conditions simultaneously for statistical purposes but also varying different process or cultivation conditions, such as filling volume, organism, and medium, within one experiment.

### Signal evaluation

Figure 1F illustrates the optical signals caused by the liquid distribution under investigation for the light transmission measurement in shake flasks. Raw signals of three aqueous polyvinylpyrrolidone (PVP) solutions of different concentrations and viscosities are shown for one rotation. Additionally, the signal of the Hall effect sensor is given as an angular position marker of the shaker movement, indicating the direction of centrifugal acceleration. The optical signals start with a horizontal course. At that point, there is no bulk liquid in front of the infrared detector. Only a thin liquid film is adhering to the hydrophilic glass wall of the flask [5, 14, 59]. Therefore, most of the emitted light can pass the liquid film and reach the detector, resulting in a high initial signal. Upon reaching the leading edge of the bulk liquid in front of the detector, an abrupt signal change occurs. The bulk liquid absorbs the light, resulting in a steep decrease of the signal. The higher the viscosity, the later the steep decrease in signal appears, indicating the leading edge of the bulk liquid *θ* (displayed for viscosities of 2.2 to 64.3 mPa·s in Fig. 1F). With increasing viscosity, the liquid film also increases in thickness, leading to a lower value of the horizontal plateau at the beginning of the signal with increasing viscosity. The arrival of the leading edge of the bulk liquid is a readily identifiable and distinct characteristic in the signal, hence it was selected as the criterion for evaluation. The intersection of two linear regressions is employed for this purpose [55]. The initial fit is implemented on the data range of the horizontal signal trajectory, when no bulk liquid is present in front of the detector. The data range corresponding to the significant signal decrease is selected for the second fit. The remaining, in parts chaotic, signal sequence, indicated in Fig. 1F by dotted lines, is not needed for the determination of the leading edge angle and can, thus, be disregarded.

## Methods

### Shake flask preparation

Glass shake flasks of the type “Erlenmeyer flasks DURAN^®^ Narrow neck” with 250 mL volume and a beaded rim (neck width of 34 mm, article number C137.1, Schott AG, Mainz, Germany) were used in this study. A new set of flasks was purchased and only used for ViMOS experiments to prevent optical distortions caused by surface scratches. The graduation on the shake flasks was eliminated through etching, and the surface was subsequently polished (Aachener Quarz-Glas Technologie Heinrich, Aachen, Germany). For subsequent experiments, to enhance the hydrophilic characteristics of the glass surface, clean and dry 250 mL Erlenmeyer shake flasks were subjected to boiling in 20 % (w/w) nitric acid (> 65 %, Carl Roth GmbH, Karlsruhe, Germany) for 45 min under a fume hood. Boiling stones (1692.1, Carl Roth GmbH, Karlsruhe, Germany) were utilized to prevent boiling retardation. The cooled shake flasks were meticulously cleaned with deionized water and subsequently dried in a drying chamber at 80 °C for 24 h.

Photographs of liquid movements in shake flasks were captured using a rotating camera as described by Seletzky et al. (2006) [40, 62]. A GoPro HERO4 black action camera (GoPro, San Mateo, CA, USA) was utilized in place of the VCAM-110 camera (Phytec^®^ Technologie Holding AG, Mainz, Germany).

### Offline viscosity measurements and viscous model liquids

Viscous flow behavior of the model liquids or cultivation broths was analyzed using a MCR 301 rheometer (Anton Paar, Stuttgart, Germany), equipped with a cone (CP50-0.5/TG, cone truncation 54 µM, cone angle 0.467°) in a range of shear rates between 10 s^− 1^ and 5000 s^− 1^ at 30 °C. The software Rheoplus/32 V3.40 (Anton Paar, Stuttgart, Germany) was used for data evaluation. Effective shear rates and corresponding apparent viscosity values for non-Newtonian fluids were determined in accordance with Giese et al. (2014) [15]. Aqueous polyvinylpyrrolidone (PVP, (C_6_H_9_NO)_n_) (Luviskol K90, BASF, Ludwigshafen, Germany) solutions with varying concentrations were used as viscous model liquids. A 15 % (w/w) stock solution was prepared with deionized water preheated to 60 °C. The solution was mixed on a magnetic stirrer with heater for about two days at 60 °C until the PVP powder was completely dissolved. Dilution series were prepared with deionized water and stored at 30 °C. The viscous flow behavior of the solutions was examined before each experiment using the MCR 301 rheometer. As aqueous PVP solutions exhibit shear thinning characteristics at elevated concentrations [55], effective shear rates and resulting apparent viscosity values were determined in accordance with Giese et al. (2014) [15]. Shear rate dependency of diluted PVP solutions was investigated before and is given in Supplementary Figure S4 [55].

### Microbial cultivation conditions

Respiration activity of all cultivations (precultures and main cultures) was online monitored using the Respiratory Activity MOnitoring System (RAMOS), developed by Anderlei et al. (2001) [8, 9]. This technology provides online data regarding the transfer rates of oxygen and carbon dioxide, as well as the respiratory quotient. For sampling, conventional shake flasks were operated in parallel under identical conditions. These “offline” shake flasks were utilized solely for sampling and were not reused in the experiment. All chemicals used in the media formulations were of analytical grade and purchased from Sigma Aldrich Co. LLC. (St. Louis, MO, USA), Merck KGaA (Darmstadt, Germany) or Carl Roth GmbH (Karlsruhe, Germany).

*Paenibacillus polymyxa* (DSM 365) was cultivated in MM1P100 medium (casein peptone 5.00 g/L, magnesium sulfate heptahydrate 1.33 g/L, potassium dihydrogen phosphate 1.67 g/L, calcium chloride dihydrate 0.05 g/L, vitamin solution RPMI 1640 1x, trace element solution (1000x): iron (II) sulfate heptahydrate 2.5 g/L, sodium tartrate dihydrate 2.1 g/L, manganese (II) chloride tetrahydrate 1.8 g/L, cobalt (II) chloride hexahydrate 0.075 g/L, copper (II) sulfate heptahydrate 0.031 g/L, boric acid 0.258 g/L, sodium molybdate 0.023 g/L, zinc chloride 0.021 g/L) [66] with 17.5 g/L glucose in 250 mL shake flasks at 200 rpm, 30 mL filling volume, 30 °C and a shaking diameter of 50 mm. All components of the MM1P100 medium were prepared individually as stock solutions. Prior to sterile filtering, the pH-value of the KH_2_PO_4_ solution was adjusted to 7.0 by adding potassium hydroxide. The trace element solution was also sterile filtered through a filter having a pore size of 0.2 μm, while the vitamin solution was purchased in aseptical form. All other components were autoclaved for 21 min at 121 °C for sterilization. The individual medium components were mixed directly before the start of the experiment. The initial pH-value of the medium was aseptically adjusted to 7.0. Precultures of *P. polymyxa* were inoculated with 0.75 mL of cryo culture (stored at -80 °C) per 30 mL MM1P100 medium. The main cultures were inoculated with an initial optical density at 600 nm (*OD*_*600*_) of 0.1 derived from the precultures harvested during the exponential growth phase. Exponential growth was determined from the respiration activity [67].

*Xanthomonas campestris pv. campestris* B100 was cultivated in a two-stage fermentation process. Initially, 40 mL of preculture (YM medium: glucose 10 g/L, yeast extract 3 g/L, malt extract 3 g/L, peptone 5 g/L, MOPS 30 g/L [68]) was inoculated with 1 mL of cryo culture (preserved at -80 °C). Precultures were carried out in 250 mL shake flasks with a filling volume of 20 mL, a shaking frequency of 300 rpm at a shaking diameter of 50 mm and a temperature of 30 °C for 19–24 h. Main cultures were inoculated from the preculture with an initial *OD*_*600*_ of 0.5. For the main cultures, GY medium (glucose 25 g/L, yeast extract 25 g/L, potassium dihydrogen phosphate 2 g/L, magnesium sulfate heptahydrate 0.205 g/L, MOPS 30 g/L) was used. All other shaking parameters corresponded to those of the preculture. To prevent the Maillard reaction [69], all complex components (yeast extract, malt extract, peptone) were autoclaved separately from the other components. Therefore, both solutions (complex components, non-complex components) were prepared at a tenfold concentration. The medium used in the experiments was prepared by mixing both solutions (complex components, non-complex components) and appropriately diluting them with sterile deionized water.

The fungal strain *Trichoderma reesei* RUT-C30 (ATCC 56765) was preserved as spore suspension with a concentration of 10^8^ spores/mL at -80 °C. For liquid cultures of *T. reesei*, modified Pakula medium, originally developed by Pakula [70] and subsequently adapted by Herweg (unpublished data), was used. Pakula medium consists of a main solution with the following final medium concentrations: diammonium sulfate 3.0 g/L, potassium dihydrogen phosphate 0.4 g/L, magnesium sulfate heptahydrate 0.5 g/L, calcium chloride dehydrate 0.23 g/L, sodium chloride 0.05 g/L, urea 0.3 g/L, Tween 80 0.01 % (v/v) and 2-Morpholinoethanesulfonic acid (MES) 0.1 mol/L. Additionally, it includes a trace element solution with the following final medium concentrations: citric acid 0.45 g/L, iron (II) sulfate heptahydrate 0.5 g/L, zinc sulfate heptahydrate 0.04 g/L, copper sulfate heptahydrate 0.008 g/L, manganese sulfate heptahydrate 0.004 g/L, borate 0.002 g/L and cobalt chloride 0.0037 g/L. The main solution and the trace element solution were sterile filtered using a 0.2 μm pore size filter. The glucose solution was autoclaved at 121 °C for 21 min. The final glucose concentration of Pakula medium was set to 30 g/L. Cultivations of *T. reesei* were performed in 250 mL shake flasks with a filling volume of 20 mL, a shaking frequency of 350 rpm at a shaking diameter of 50 mm and 30 °C. For inoculation, the initial spore concentration was set to 10^6^ spores/mL. Preliminary experiments were performed using modified Pakula medium with 50 g/L glucose and 100 mM PIPPS buffer.

The maximum oxygen transfer capacity (*OTR*_*max*_) for waterlike viscosities was calculated according to Meier at al. (2016), based on the applied cultivation parameters and measured osmolality (Eq. 3) Meier at al. (2016) [71]. In this context, *Osmol* [Osmol kg^− 1^] denotes the culture osmolality, *n* [min^− 1^] represents the shaking frequency, *V*_*L*_ [mL] indicates the filling volume, *d*_*0*_ [cm] refers to the shaking diameter, *d* [mm] indicates the maximum inner shake flask diameter, *p*_*R*_ [bar] represents the reactor pressure and *y*_O2*_ [mol mol^− 1^] denotes the oxygen mole fraction in the gas phase.

3 $$\begin{array}{l}\:{OTR}_{max}=3.72\:\cdot\:\:{10}^{-7}\:\cdot\:{Osmol}^{\text{0.05}}\:\cdot\:\:{n}^{\left(1.18-\:\frac{Osmol}{10.1}\right)}\\\cdot\:\:{V}_{L}^{-0.74}\cdot\:{d}_{0}^{0.33}\cdot\:{d}^{1.88}\cdot\:{p}_{R}\cdot\:{y}_{{O}_{2}^{*}}\end{array}$$

## Results and discussion

### Influence of the shake flask orientation on the leading edge angle of the bulk liquid

**Fig. 2:**
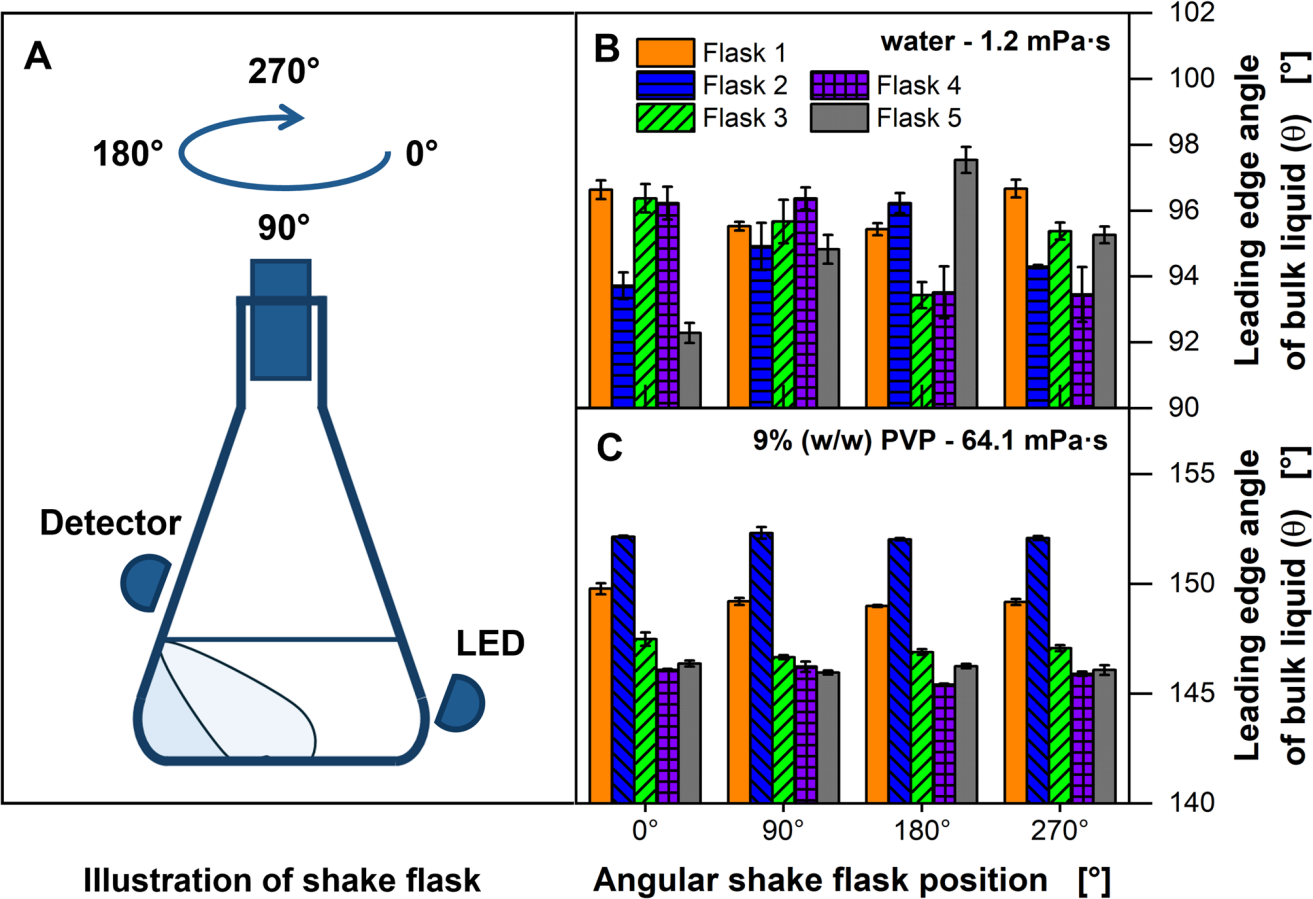
Determination of leading edge angle of bulk liquid θ by transmitted light measurement for five shake flasks with varying orientation. (**A**) Schematic representation of the 90° stepwise rotation and measurement of the shake flask along its longitudinal axis. (**B**) Shake flasks filled with water (1.2 mPa∙s). (**C**) Shake flasks filled with 9 % (w/w) PVP (64.1 mPa∙s). (**B** & **C**) LED angle: 121°, detector angle: 55°. *V*_*F*_ = 250 mL, *V*_*L*_ = 20 mL, *n* = 350 min^− 1^, *d*_*0*_ = 50 mm, *T* = 30 °C. Error bars represent the standard deviation of triplicates. Parts of this figure have been adapted from Sieben (2017) [72]

Due to their manufacturing process, shake flasks are neither perfectly rotationally symmetric nor completely identical to each other. To quantify the influence of those differences on the determination of the leading edge angle of the bulk liquid *θ*, five individual shake flasks were investigated in four angular positions (Fig. 2). Every shake flask was mounted on the measuring setup and filled with either water (Fig. 2B) or a PVP solution (Fig. 2C). While being mounted, the shake flask was rotated cautiously in steps of 90° on its longitudinal axis until it reached the initial angular position (Fig. 2A). In each position, *θ* was determined. The results show clear differences regarding the determination of *θ* for the individual shake flasks and their four angular positions. For example, shake flask 5 indicates a difference of more than five degrees in *θ*, when it is rotated from 0° to 180° for experiments with water (Fig. 2B). The standard deviation for the four angular measurements of each shake flask ranges from 0.68° (shake flask 1) to 2.15° (shake flask 5), while the standard deviation between shake flasks in the same angular position ranges from 0.63° at the 90° position to 1.94° at the 180° position. These results show that the optical differences between rotating one flask are approximately in the same order of magnitude as the optical differences between multiple shake flasks. However, when using a PVP solution with a viscosity of 64.1 mPa∙s (Fig. 2C), the fluctuations at different angular positions of a shake flask are significantly lower, compared to the difference between the five shake flasks. It is assumed that the measured differences in *θ* result from manufacturing-related differences in the thicknesses and surface irregularities of the glass wall of the shake flasks. These lead to slightly different optical conditions, when rotating a shake flask or using a different one. Alternatively, experiments were performed with plastic shake flasks (4103 − 0250, Thermo Fisher Scientific Inc., Waltham, USA). These flasks have a hydrophobic surface property. Clear leading edges could not be determined with these flasks, due to the strongly fluctuating output signals of the light detector (data not shown). Therefore, experiments were continued with glass flasks. For all calibrations, shake flask cultivations and other ViMOS measurements, only one specific glass flask was used in each position rotated to the same angular position.

### Influence of shake flask pretreatment

**Fig. 3:**
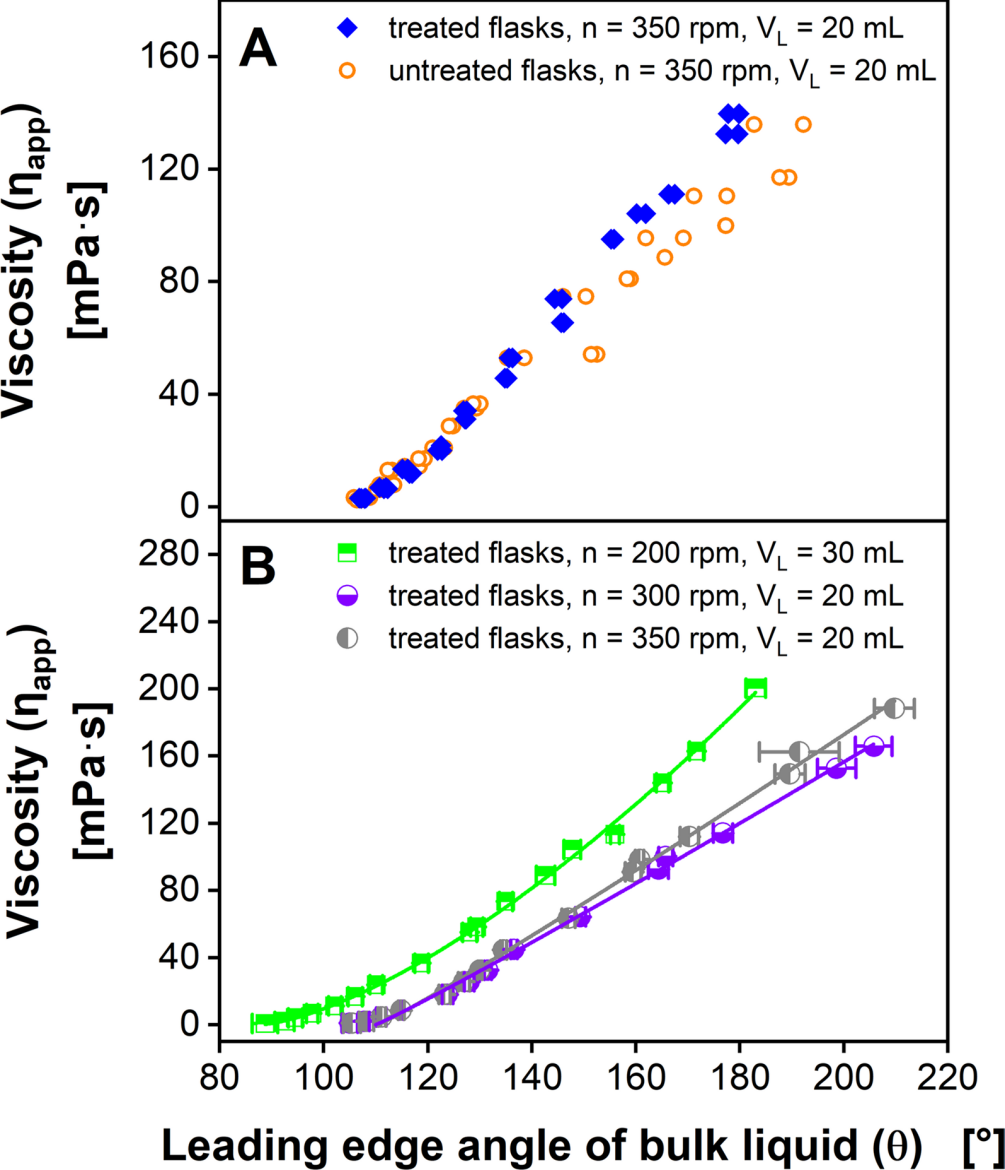
Influence of shake flask pretreatment on leading edge angle. (**A**) Influence of wetting properties of the inner flask wall on the leading edge angle of the bulk liquid *θ*. LED angle: 124°, detector angle: 55°. (**B**) Calibration of *θ* with apparent viscosity based on Eq. 4 using treated shake flasks for varying shaking frequencies *n* and filling volumes *V*_*L*_. Fit parameters for calibration functions are listed in Supplementary Table S4. PVP solutions of different concentrations, LED angle: 126°, detector angle: 53°. *V*_*F*_ = 250 mL, *d*_*0*_ = 50 mm, *T* = 30 °C. Error bars represent the standard deviations of triplicates. Parts of this figure have been adapted from Sieben (2017) [72]

As mentioned before, the use of hydrophobic plastic shake flasks resulted in an increased noise of the optical signals. Therefore, the influence of the degree of hydrophilicity of the inner shake flask wall on the leading edge angle of the bulk liquid *θ* was investigated in consecutive experiments for PVP solutions with viscosities of 3 to 140 mPa∙s (Fig. 3A). Up to a viscosity of approximately 50 mPa·s, the measured values of *θ* show a low deviation for untreated shake flasks (Fig. 3A, orange circles). However, for viscosities higher than 50 mPa·s, *θ* differs up to 15.3° for a viscosity of 54.3 mPa∙s and up to 9.5° for a viscosity of 135.7 mPa∙s. Despite the expectation that equal viscosity would correspond to an equal shift of the leading edge of the bulk liquid, the results reveal that the reproducibility of *θ* was inadequate for increased viscosities, leading to deviations between measured and calculated viscosities up to 20 % (see Supplementary Figure S5).

Borosilicate glass consists mainly of silicone dioxide and is usually hydrophilic due to hydroxyl groups on the surface. However, metal ions can replace the hydrogen atoms of the hydroxyl groups and lead to undefined wetting properties. To recover the hydrophilic properties and create identical conditions in all flasks, the flasks were pretreated by boiling with nitric acid for 45 min. Experiments were then repeated with the hydrophilized, “pretreated” flasks (Fig. 3A, filled blue diamonds). After pretreatment, the deviation of *θ* for the same viscosity was clearly reduced, showing the largest differences of *θ* up to 1.8° for a viscosity of 104.1 mPa∙s and 2.2° for a viscosity of 132.5 mPa∙s. The slight differences, which still occur, might be attributed to the different wall thickness of the different flasks. However, a flask-specific offset could be determined, enabling a zero calibration with respect to the cultivation processes (Supplementary Figure S6). To guarantee reliable conditions for calibrations, shake flask cultivations and other ViMOS measurements, the shake flasks were regularly treated with 20 % (w/w) boiling nitric acid for 45 min as described before.

### Application of viscosity online monitoring for bacterial and fungal cultivations

Previous studies have used linear fits to correlate the angle of the leading edge of the bulk liquid *θ* and the apparent viscosity of the culture broth *η*_*app*_ [55, 56]. This is a feasible option for a narrow range of apparent viscosities. To monitor microbial cultivations ranging from waterlike viscosities of 0.9 mPa·s up to 150 mPa·s and higher, linear fits do not represent the whole range adequately. Although linear fitting will yield high coefficients of determination (R² ≥ 0.976), viscosities below 10 mPa·s will be underestimated and viscosities between 10 and 70 mPa·s might be slightly overestimated (Supplementary Figure S7). Therefore, calibrations of *η*_*app*_ against *θ* were performed using a root function with the fitting parameters *a*, *b* and *c*, given in Eq. 4 (Fig. 3B).

4 $$\:{\eta\:}_{app}=\sqrt[b]{(\theta\:-c)/a)}$$

Since alterations of the filling volume, shaking frequency and shaking diameter influence the calibration function [55, 56], a new calibration must be carried out for each combination of shaking parameters that is chosen for a biological cultivation. Obtained fit parameters are listed in Supplementary Table S4. Considering the three chosen combinations of shaking parameters, viscosities above 126 to 147 mPa·s lead to a *Ph* below the critical Phase number *Ph*_*crit*_ according to Büchs et al. (2000) [2] of 1.26 but still above the critical Phase number *Ph*_*crit*_ according to Azizan et al. (2019) [60] of 0.91. After successful calibration, viscosity can be calculated with the measured leading edge angle *θ* and the system can be used for monitoring the viscosity of a fermentation broth in the calibrated viscosity range. While in this work Erlenmeyer flasks manufactured by Schott AG were used for the calibration and preparation of ViMOS measurements, future works might focus on a comparison to shake flasks of other manufacturers such as Kimble Chase or Corning to assess transferability of the results.

To demonstrate the benefits for process monitoring by combining the RAMOS and the ViMOS technology, three different viscous model cultivation systems were tested in shake flasks, enabling a periodical measurement of the oxygen transfer rate (*OTR*) as well as the apparent viscosity *η*_*app*_ [8, 9, 55]. The tested cultivations cover small viscosity increases due to exopolysaccharide production of xanthan by *X. campestris* [20], large viscosity increases due to the secretion of structurally diverse exopolysaccharides by *P. polymyxa* [73] as well as viscosity changes due to filamentous growth of the fungus *T. reesei* [74].

For validation, the online viscosity signal of the bacterial cultures was compared to offline measured viscosity values, using a conventional cone-plate rheometer. As the culture broths of all shake flask cultivations exhibited non-Newtonian, shear-thinning properties (Ostwald–de Waele relationship), the apparent viscosity for the effective shear rate is given according to Giese et al. (2014) [15]. In parallel, identical cultivations were performed in regular shake flasks without online monitoring, to exclude differences of either biological or physical nature between cultures in ViMOS flasks and cultures in regular shake flasks. Samples were taken from ViMOS cultures as well as regular shake flask cultures and subjected to offline analysis. Since the online viscosity signal strongly depends on the filling volume [55], the signal of the ViMOS shake flasks used for sampling was no longer considered after the time of sampling. Also, none of the offline shake flasks were returned to the cultivation after being used for sampling.

### Cultivation of *Xanthomonas campestris* with small viscosity changes

**Fig. 4:**
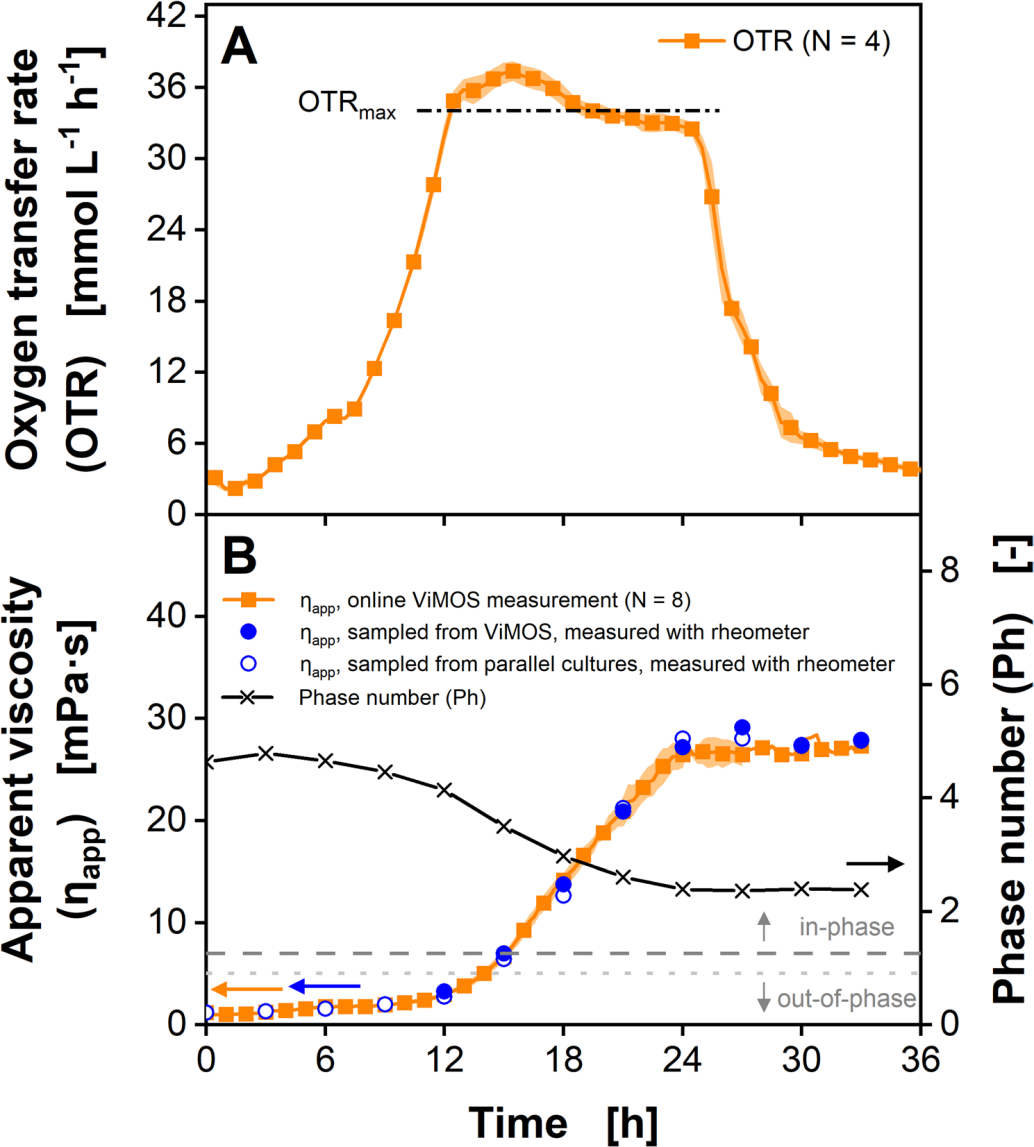
Cultivation of *Xanthomonas campestris pv. campestris* B100 in GY medium with simultaneous online monitoring of the oxygen transfer rate and apparent viscosity. (**A**) Oxygen transfer rates. For reasons of clarity, only every second data point is shown. Maximum oxygen transfer capacity *OTR*_*max*_ is calculated according to Eq. 3 (Meier et al., 2016) [71]. Average of four biological replicates is shown. Transparent shadow represents standard deviation. (**B**) Online viscosity signal and offline measured viscosity values. Every 15 min, *θ* was determined from 100 rotations and converted into a viscosity value by using the calibration function (Eq. 4). Average of two to eight biological replicates is shown. Transparent shadow represents standard deviation. For reasons of clarity, only every fourth point is shown for the online signals. For offline measurements, samples were taken from both ViMOS and parallel regular shake flasks, incubated in a different shaker. The Phase number *Ph* is calculated based on measured viscosity according to Eq. 1 (Büchs et al. 2000) [2]. The dark grey dashed line marks the critical *Ph* of 1.26 according to Büchs et al. (2000) [2]. The light grey dotted line marks an alternative critical *Ph* according to Azizan et al. (2019) [60]. *V*_*F*_ = 250 mL, *V*_*L*_ = 20 mL, *n* = 300 min^− 1^, *d*_*0*_ = 50 mm, *T* = 30 °C. Parts of this figure have been adapted from Sieben (2017) [72]

*X. campestris* is a gram-negative bacterium known to cause a variety of plant infections, including the black rot [75]. As a natural producer of xanthan-gum, this organism is commercially used for exopolysaccharide production, which leads to a viscous fermentation broth [20, 76]. Therefore, it has often been used to showcase new methods for viscosity monitoring [15, 50, 56, 57]. A *X. campestris* shake flask cultivation on complex GY medium was online monitored via the RAMOS and the ViMOS (Fig. 4, individual replicates shown in Supplementary Figure S8). To validate the apparent viscosity *η*_*app*_ values of the ViMOS, samples were taken every 3 h and measured on a cone-plate rheometer (number of replicates for online viscosity measurement in Fig. 4B decreases by one, each time a flask was harvested for offline viscosity measurement). The matching courses of the *OTR* and the *η*_*app*_ values demonstrate an excellent reproducibility of both online monitoring techniques. Due to the high inoculation density using an exponentially growing pre-culture, a short lag phase below 1.5 h was observed.

Until 12 h of cultivation, the *OTR* increases to 34 mmol/L/h and indicates a non-oxygen limited exponential growth on glucose, while the apparent viscosity *η*_*app*_ only increased from 0.8 to 3.0 mPa·s. Between 12.5 and 24.5 h of cultivation time, an oxygen limitation was reached, indicated by an *OTR* plateau and the absolute value of the plateau being close to the maximum oxygen transfer capacity *OTR*_*max*_ of 34 mmol/L/h, which was calculated according to Meier at al. (2016) at an osmolality for the GY medium of 0.481 osmol/kg [8, 9, 71]. Interestingly, the *OTR* slightly increased until its peak at 16 h and an *OTR* of 38 mmol/L/h. This effect was initially predicted by Maier et al. (2004) [5] and further investigated by Giese et al. (2014) [14]. The observed *OTR* level above *OTR*_*max*_ is caused by an apparent viscosity *η*_*app*_ level slightly below 9 mPa·s. With increasing viscosities, a thicker liquid film is formed at the hydrophilic shake flask wall, resulting in an enhanced diffusion of oxygen into the liquid film and an elevated maximum oxygen transfer capacity *OTR*_*max*_ [14]. However, at viscosities higher than approximately 10 mPa·s, the *OTR*_*max*_ decreases, due to reduced diffusion and mass transfer. As a result, the *OTR* decreases between 16 und 25 h of cultivation, while the apparent viscosity *η*_*app*_ increases from 9 to 28 mPa·s. After 25 h, the *OTR* dropped, most likely due to glucose depletion, while the apparent viscosity *η*_*app*_ remained constant.

During the entire *X. campestris* shake flask cultivation, the apparent viscosity *η*_*app*_ values, online monitored using the ViMOS as well as cultivated in regular shake flasks, matched the apparent viscosity *η*_*app*_ measured offline, using the cone-plate rheometer (Fig. 4B). As indicated by the Phase number *Ph* > 2.3, the culture liquid remains in-phase during the entire cultivation time. These results are consistent with the observations by Ladner et al. (2019) [56] and demonstrate the improved reproducibility of the online viscosity measurement due to the shake flask pretreatment, the root function calibration method (Eq. 4) and offset correction [15, 24, 54]. As the ViMOS does not require using fluorescent oxygen-sensitive nanoparticles, this method is less expensive, compared to the approach by Ladner et al. (2019) [56]. Furthermore, the ViMOS viscosity monitoring of *X. campestris* shake flask cultures is less labor-intensive, compared to the specific power input measurement demonstrated by Giese et al. (2014) [77].

### Cultivation of *Paenibacillus polymyxa* with large viscosity changes

**Fig. 5:**
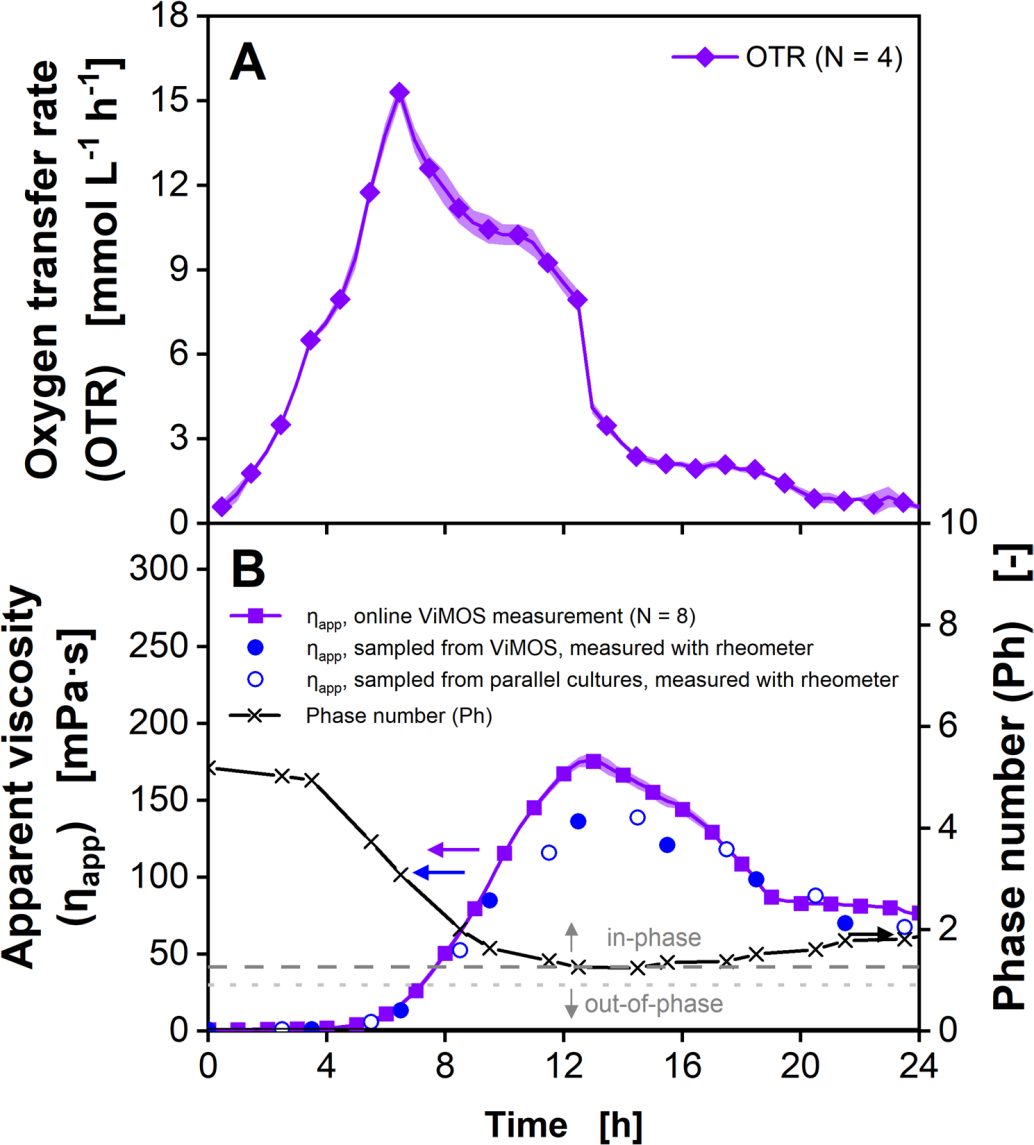
Cultivation of *Paenibacillus polymyxa* DSM 365 in MM1P100 medium with simultaneous online monitoring of the oxygen transfer rate and apparent viscosity. (**A**) Oxygen transfer rates. For reasons of clarity, only every second data point is shown. Average of four biological replicates is shown. Transparent shadow represents standard deviation. (**B**) Online viscosity signal and offline measured viscosity values. Every 15 min, *θ* was determined from 100 rotations and converted into a viscosity value by using the calibration function (Eq. 4). Average of two to eight biological replicates is shown. Transparent shadow represents standard deviation. For reasons of clarity, only every fourth data point is shown for the online signals. For offline measurements, samples were taken from both ViMOS and parallel regular shake flasks, incubated in a different shaker. The Phase number *Ph* is calculated based on measured viscosity according to Eq. 1 (Büchs et al. 2000) [2]. The dark grey dashed line marks the critical *Ph* of 1.26 according to Büchs et al. (2000) [2]. The light grey dotted line marks an alternative critical *Ph* according to Aizizan et al. (2019) [60]. *V*_*F*_ = 250 mL, *V*_*L*_ = 30 mL, *n* = 200 min^− 1^, *d*_*0*_ = 50 mm, *T* = 30 °C. Parts of this figure have been adapted from Sieben (2017) [72]

Recently, Halmschlag et al. (2023) and Hoffmann et al. (2023) applied a combined RAMOS and ViMOS monitoring to investigate *Bacillus subtilis* strains genetically modified to produce the biopolymer poly-γ-glutamic acid (γ-PGA) [17, 18, 58]. During their experiments, the *B. subtilis* shake flask cultures reached viscosities up to 42 mPa·s. To validate the ViMOS method for higher viscosities, shake flask cultures of *P. polymyxa*, formerly known as *Bacillus polymyxa*, were monitored using the RAMOS and the ViMOS (Fig. 5, individual replicates shown in Supplementary Figure S9). *P. polymyxa* is a gram-positive, endospore forming bacterium that produces exopolysaccharides during nitrogen limitation [78]. Being important for biofilm formation [79] and plant root colonization [80], these exopolysaccharides lead to highly viscous fermentation broths.

Without any visible lag-phase, the *OTR* of the *P. polymyxa* culture indicates unlimited exponential growth, until the *OTR* reaches a peak value of about 16 mmol/L/h after 6.5 h of cultivation (Fig. 5A). During the next 6 h, the *OTR* decreases to about 8 mmol/L/h, which indicates a secondary substrate limitation [81]. Subsequently, the *OTR* abruptly drops to 3.5 mmol/L/h between 12.5 h and 13.5 h. During the first 4 h, the apparent viscosity *η*_*app*_ only shows a small increase to 2.3 mPa·s. However, from 4 h until 12.5 h, the apparent viscosity *η*_*app*_ exponentially increases to an online monitored value of 176 mPa·s, while the culture sample measured on the cone-plate rheometer only shows a value of 136 mPa·s (Fig. 5B). It is assumed that the apparent viscosity *η*_*app*_ difference of 40 mPa·s between the ViMOS and the rheometer measurement are due to a gradual transition to out-of-phase conditions, as the Phase number *Ph* approaches a value 1.26 at 12.5 h. After 12.5 h, the apparent viscosity *η*_*app*_ starts to decrease down to 88 mPa·s at 19 h. It was described by Lee et al. (1997) [82] that the exopolysaccharide produced by *P. polymyxa* is degraded upon nitrogen limitation.

This result was confirmed by results of Rütering et al. (2016) [66], which led to the assumption that nitrogen might have been exhausted at 12.5 h, leading to an *OTR* drop as well as to the degradation of the exopolysaccharide and consumption as carbon source by *P. polymyxa.* Similar to the *X. campestris* cultivation, the matching *OTR* and *η*_*app*_ trajectories of all *P. polymyxa* cultures demonstrate the reproducibility of the RAMOS and ViMOS online monitoring.

During the *P. polymyxa* shake flask cultivation, apparent viscosity *η*_*app*_ values determined using the ViMOS and a cone-plate rheometer measurement showed matching *η*_*app*_ values up to 118 mPa·s and a Phase number above 1.37. The online monitored and offline measured viscosity values matched before and after the viscosity peak, up to 9.5 h and after 17.5 h of cultivation time. For higher apparent viscosities, differences between 20 mPa·s (at 14.5 h) and 40 mPa·s (at 12.5 h) were observed. This discrepancy could have been caused by foam formation interfering with the detection of the leading edge of the bulk liquid. In contrast, Dinter et al. (2024) were able to demonstrate online viscosity measurements based on the bulk liquid angle during a *P. polymyxa* shake flask cultivation for viscosities between 100 and 150 mPa·s [57]. For viscosities above 150 mPa·s, Dinter et al. (2024) also reported the formation of foam [57]. In preliminary experiments, Michaela Sieben (2017) showed matching ViMOS online viscosity values and offline measured viscosities up to 250 mPa·s [72]. During the monitoring of biopolymer production of *X. campestris* (Fig. 4) and *P. polymyxa* (Fig. 5) cultivations, apparent viscosity determined online using the ViMOS matched apparent viscosity measured using a cone-plate rheometer up to 180 mPa·s with an accuracy of ± 20 % (see Supplementary Figure S10). However, further experiments at viscosities above 120 mPa·s and different culture conditions, using a non-foaming microbial system are needed. For now, the ViMOS should only be used to monitor cultivations of apparent viscosities *η*_*app*_ below 118 mPa·s and *Ph* above 1.37, until measurements for higher viscosities are successfully validated.

### Cultivation of the filamentous fungus *Trichoderma reesei*

**Fig. 6:**
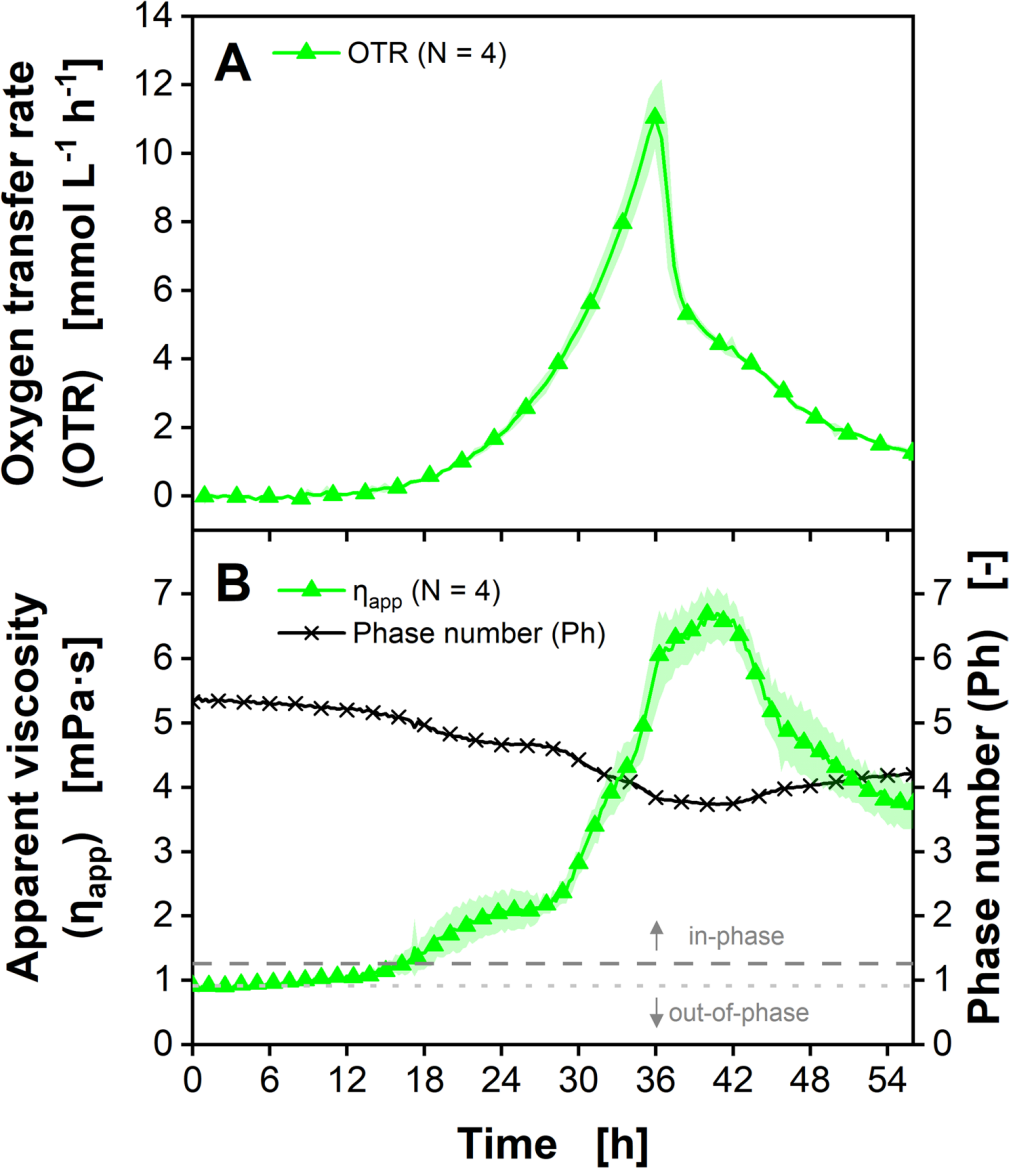
Cultivation of *Trichoderma reesei* RUT-C30 in modified Pakula medium with simultaneous online monitoring of the oxygen transfer rate and apparent viscosity. (**A**) Oxygen transfer rates. For reasons of clarity, only every second data point is shown. Average of four biological replicates is shown. Transparent shadow represents standard deviation. (**B**) Online viscosity signal. Every 15 min *θ* was determined from 200 rotations and converted into a viscosity value by using the calibration function (Eq. 4). Average of four biological replicates is shown. Transparent shadow represents standard deviation. For reasons of clarity, only every eighth data point is shown. The Phase number *Ph* is calculated based on measured online viscosity according to Eq. 1. For reasons of clarity, only every eighth data point is shown. The dark grey dashed line marks the critical *Ph* of 1.26 according to Büchs et al. (2000) [2]. The light grey dotted line marks an alternative critical *Ph* according to Azizan et al. (2019) [60]. *V*_*F*_ = 250 mL, *V*_*L*_ = 20 mL, *n* = 350 min^− 1^, *d*_*0*_ = 50 mm, *T* = 30 °C

Accurately monitoring the apparent viscosity of filamentous bacterial or fungal cultures in shake flasks is challenging, since pelleted morphology as well as filamentous growth complicate performing reliable measurements [27, 83, 84]. Especially morphological changes during the cultivation might require using multiple rheometer methods to avoid sedimentation, wall slipping effects or disruption of the mycelial structure due to shear stress [27, 83, 84]. *Trichoderma reesei* is a mesophilic and filamentous growing fungus. Its cellulase and hemicellulase production capabilities are of industrial relevance in the conversion of plant biomass (cellulose) to glucose [85, 86]. In contrast to the biopolymer producing cultivations of *X. campestris* and *P. polymyxa* described above, the *T. reesei* cultivation shown in Fig. 6 (individual replicates shown in Supplementary Figure S11) exhibits an increase in viscosity caused by the filamentous growth of the fungus itself. During the first 12 to 18 h, *T. reesei* shows a long lag phase, indicated by the *OTR* being almost zero (Fig. 6A). During this time, the apparent viscosity *η*_*app*_ only marginally increases from roughly 0.9 to 1.2 mPa·s (Fig. 6B). This might already indicate first morphological changes or mycelial growth. Between 12 and 18 h, the *OTR* starts to rise to 12 mmol/L/h, until approximately 36 h. Then, the *OTR* steeply drops to 6 mmol/L/h, indicating the depletion of the carbon source. During the following 18 h, the *OTR* and decreases to 2 mmol/L/h. Between 18 and 30 h, the *η*_*app*_ roughly doubles to 2.5 mPa·s, before steeply increasing to 6.0 mPa·s at 36 h. After the *OTR* drop at 36 h, the viscosity remains roughly constant until 42 h, before decreasing below 4 mPa·s, probably due to mycelial disintegration caused by the carbon source depletion. As indicated by the Phase number *Ph* above 1.26, the culture stays in-phase during the whole cultivation time. Unfortunately, offline validation of the apparent viscosity could not be performed, as the available a cone-plate or plate-plate rheometers is not suited for particulate systems, such as mycelial fermentation broths (see Supplementary Figure S12 & S13). Additional shake flask cultivations of *T. reesei* using up to 150 g/L α-cellulose as substrate resulted in similar viscosity developments as α-cellulose did not raise the viscosity of the culture broth (data not shown).

The relationship between *OTR* and apparent viscosity is different for each of the three chosen microorganisms. During the cultivation of *X. campestris* (Fig. 4), the biopolymer xanthan is produced during the late growth phase and during oxygen limitation. Therefore, apparent viscosity is increasing from 12 h to 24 h. After 24 h, the *OTR* decreases, indicating glucose depletion. As Xanthan is not degraded by *X. campestris*, the apparent viscosity remains constant. The production of the exopolysaccharides by *P. polymyxa* is triggered by a nitrogen limitation, indicated by a decreasing *OTR* between 6 h and 12 h (Fig. 5). After 12 h, the primary substrate is depleted. This is when the degradation of the exopolysaccharides starts and apparent viscosity decreases. During the cultivation of *T. reesei* (Fig. 6), the apparent viscosity is not influenced by the production of a biopolymer, but the filamentous biomass itself. That is why apparent viscosity increases during the fungal growth phase from roughly 12 h to 36 h and decreases afterwards.

Although the culture viscosity plays a crucial role for mixing and mass transfer of filamentous cultures, it is rarely measured in *T. reesei* shake flask cultivations [36, 87, 88, 89, 90]. If viscosity is measured, this is a laborious procedure, as different rheometer setups are required for different cultivation phases. At the beginning of the cultivation, when the viscosity is still low and mycelial or pellet particles still small, a plate-plate rheometer setup can be used [27, 77, 91]. For larger pellets or mycelial structures, a vane-cup rheometer is necessary, which requires high sample volumes of 15 mL or more.

It allows for only one measurement replicate, as mycelia and pellets may disintegrate at higher shear rates applied during the measurement [27, 77]. Unfortunately, vane-cup rheometers are further restricted to measurements in the laminar or early transitional flow regime [27]. Giese et al. (2014) measured the volumetric power input of a *T. reesei* shake flask culture, which can be used to calculate the apparent culture viscosity. However, this approach is laborious and allows for only one replicate to be measured per cultivation [77]. Therefore, the ViMOS represents a currently unique possibility for online viscosity monitoring in not only one, but up to eight shake flasks in parallel.

## Conclusions

This work demonstrates the reproducible application of the ViMOS technology for online viscosity monitoring of bacterial biopolymer production up to viscosities of 120 mPa·s and for filamentous fungi in up to eight parallel shake flasks. The combination with *OTR* monitoring via a RAMOS device allows detection of microbial growth phases, oxygen limitations, biopolymer production and degradation as well as the growth of filamentous cultures.

For reliable viscosity measurements, it is crucial to keep a consistent shake flask orientation and to regularly perform a shake flask pretreatment, to guarantee hydrophilic shake flask surface properties. When investigating microbial cultures with changing viscosities, a linear calibration function of apparent viscosity against the leading edge angle of the bulk liquid is no longer sufficient. Three fitting parameters describing the observed square root relationship between apparent viscosity and the leading edge angle must be determined for each combination of shaking frequency, diameter and filling volume. As this introduces practical limitations for other users, developing generalized calibration methods or a computational tool for predicting parameters under new conditions would facilitate the adoption of the ViMOS. Modelling approaches based on the already available parameters sets generated at different operating conditions might help to generate calibration functions for novel cultivation parameter combinations. First steps were already taken by Dinter et al. (2024) by CFD modelling viscous fluids in shake flasks [42, 61].

Further experiments are required to investigate the reason for the discrepancies of ViMOS and rheometer measurements above viscosities of 120 mPa·s. The detection of the leading edge angle *θ* is dependent on an abrupt signal drop after a clear baseline signal (Fig. 1F). If foam formation interferes with the baseline signal or introduces noise during the expected signal drop, the leading edge angle cannot be detected properly. As a similar system called ShakeVisc has been validated up to 150 mPa·s [57], further experiments using non-foaming microbial cultures should be performed. Since it is assumed that foam formation was caused by out-of-phase effects, out-of-phase conditions must be investigated separately. As the current viscosity calculation is based on the culture filling volume, monitoring of longer cultivations must consider the loss of liquid due to evaporation.

The cultivation and viscosity monitoring of *T. reesei* is only the first step to establishing the ViMOS as a tool for the investigation of growth and morphological changes of filamentous cultures. As next steps, a detailed monitoring of biomass concentration, cell morphology and rheological properties of different filamentous organisms will reveal to which extend the ViMOS can be used for morphology monitoring. Furthermore, it is critical to provide reliable offline rheometer measurements for the validation of the shear rate dependent viscosity of filamentous cultures [27, 84].

In conclusion, applying the ViMOS technology offers an inexpensive method to monitor the rheological behavior of the fermentation broth, to gain a better understanding about the dynamically changing influences of apparent viscosity on mixing, gas/liquid mass transfer and power input. An interruption of the cultivation process for time-consuming sampling and offline analysis can be avoided and screening conditions in shake flasks as well as the subsequent scale-up can be improved.

## Supplementary Information

Below is the link to the electronic supplementary material.

Supplementary Material 1

## Data Availability

The datasets used and/or analyzed during the current study are available from the corresponding author on reasonable request.

## Nomenclature

η [mPa·s]: Dynamic viscosity
η_*app*_ [mPa·s]: Apparent viscosity
η_*crit*_ [mPa·s]: Critical viscosity
θ [°]: Leading edge angle of the bulk liquid
OTR $\left[\frac{mmol}{L\cdot\:\text{h}}\right]$: Oxygen transfer rate
OTR_*max*_$\left[\frac{mmol}{L\cdot\:\text{h}}\right]$: Maximum oxygen transfer capacity
$$\:n\:\left[\frac{1}{\text{m}\text{i}\text{n}}\right]$$: Shaking frequency
*V*_*L*_ [mL]: Filling volume
*d*_*0*_ [mm]: Shaking diameter
*d* [mm]: Maximum inner shake flask diameter
*T* [°C]: Temperature
*Fr*_*a*_ [−]: Axial Froude number
*Ph* [−]: Phase number
*Ph*_*crit*_ [−]: Critical Phase number
*OD*_*600*_ [−]: Optical density measured at a wavelength of 600 nm
*a* [−]: Fit parameter
*b* [−]: Fit parameter
*c* [−]: Fit parameter
