# Supplementary material for: Simultaneous online monitoring of viscosity and oxygen transfer rate in shake flask cultures
Source: J Biol Eng. 2025 Aug 22;19:77. doi: 10.1186/s13036-025-00552-6 (PMC12374283; doi:10.1186/s13036-025-00552-6)
Supplement: Supplementary file 1 — Supplementary Material 1 [file 13036_2025_552_MOESM1_ESM.pdf]

Supplementary Material

Simultaneous online monitoring of viscosity and oxygen transfer rate in shake flask cultures

Hanke, René[1*]

Sieben, Michaela[1*]

Finger, Maurice[1]

Schnoor, Kilian[1]

Jeßberger, Simon[1]

Weyand, Julia[1]

de la Fuente, Lluìs Coloma[1]

Mann, Marcel[1]

Azizan, Amizon[2]

Kosfeld, Udo[1]

Büchs, Jochen[1*]

[1]AVT.BioVT - Chair of Biochemical Engineering, Rheinisch-Westfälische Technische Hochschule (RWTH) Aachen University, Aachen, Germany

[2] Faculty of Chemical Engineering, Universiti Teknologi MARA, 40450 Shah Alam, Selangor, Malaysia

[*]René Hanke and Michaela Sieben contributed equally to this work.

Correspondence: Prof. Jochen Büchs (jochen.buechs@avt.rwth-aachen.de). AVT.BioVT - Chair of Biochemical Engineering, Rheinisch-Westfälische Technische Hochschule (RWTH) Aachen University, Aachen, Germany

Table S1: Comparison of published methods for online viscosity measurement.

		Lotter et al. (2004) (54)	Ladner et al. (2019) (56)	Dinter et al. (2024) (57)	ViMOS (this work)
**Measurement characteristics**	**Type of measurement**	Torque measurement	Optical, backscatter	Optical, backscatter	Optical, transmission
	**Recorded parameter**	Specific power input	Phase angle of bulk liquid	Phase angle of bulk liquid	Phase angle of bulk liquid
	**Special equipment needed**	Torque sensor	Hall effect sensor, custom LED and detector	ShakeVisc module (including LED, detector and acceleration sensor)	Hall effect sensor, custom LED and detector
	**Calibration needed**	Yes	Yes	Yes	Yes
	**prerequisite for measurement**	Warming up for 24 h	Yes, oxygen-sensitive nanoparticles	Nanoparticles optional	No
**Constraints for measured fluids**	**Dark fluids can be measured**	Yes	More nanoparticles needed for darker fluids	Yes	Yes
	**Fluorescent fluids can be measured**	Yes	Yes	Yes	Yes
	**Low liquid volumes possible**	Low liquid volumes result in lower accuracy	Yes	Yes	Yes
**Operational constraints**	**Shake flask sizes tested**	From 250 mL to 2000 mL	250 mL	250 mL	250 mL
	**Number of flasks needed to measure one condition**	9 flasks of 500 mL or 14 flasks of 250 mL volume	One	One	One
	**Flexibility**	Only one condition per experiment	Type of fluid and filling volume individual for each flask	Type of fluid and filling volume individual for each flask	Type of fluid and filling volume individual for each flask
**Further remarks**	**Combination with RAMOS/TOM**	No	Yes	Yes	Yes
	**Combination with pH or DOT measurement**	Not tested yet	Yes	Yes	Not tested yet
	**Additional comments**	Laborious and noisy method	Additional expenses for nanoparticles	Viscosity determination without nanoparticles only possible after sufficient biomass is formed (OD_600 > 3)	Construction of custom-made measurement setup requires special expertise

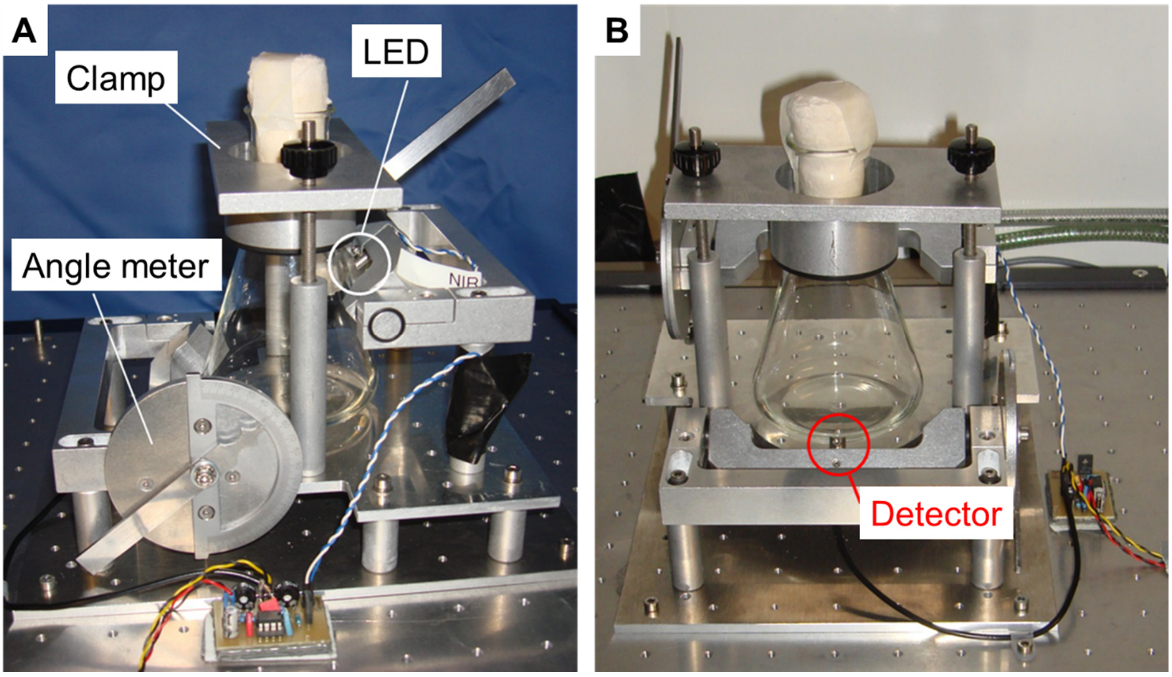

Figure S1: Photos of the transmitted light measuring set-up for one shake flask. Light source and detector are on opposite sides of the shake flask. (**A**) Side view (**B**) Side view rotated by 90°. Figure adapted from Sieben (2017) (69).

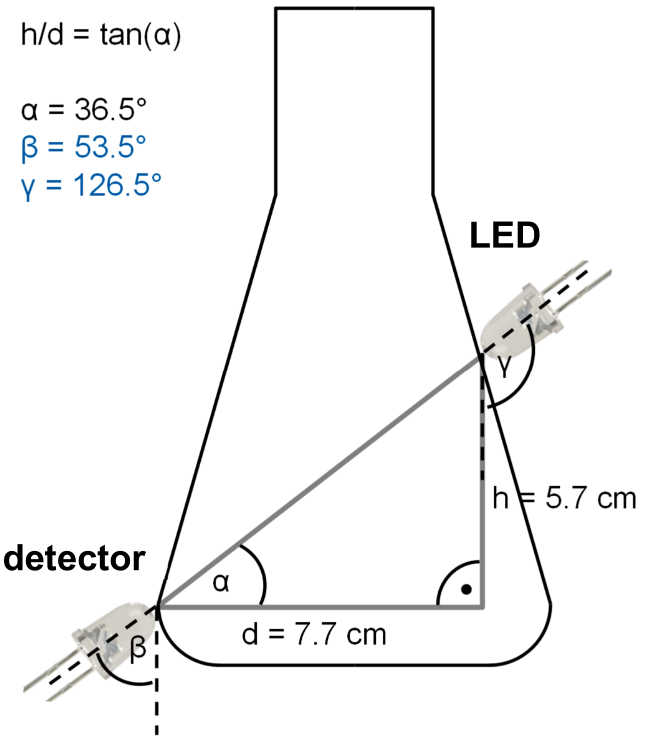

Figure S2: Mathematical approach to identify the best orientation for the LED and the detector with respect to the longitudinal axis of the shake flask. Figure adapted from Sieben (2017) (69).

Table S2: Influence of LED and detector angle setting on signal intensity of water. V_K = 250 mL, Flask No. 6, V_L = 10 mL, n = 350 rpm, d_0 = 50 mm, T = 30°C, H_2O. First row: LED angles, first column: detector angles. The signal intensity was determined by averaging the values of the horizontal signal sequence of the raw signal. The results show the relative signal intensity with respect to the highest signal intensity, from the lowest signal intensity in red to the highest signal intensity in green. Grey fields indicate angle positions, for which no measurement was performed. Table adapted from Sieben (2017) (69).

[°]	123	124	125	126	127	128	129	130
52	0.144			0.926	0.816			0.889
53	0.162		0.905	0.943	0.829		0.984	0.914
54	0.186	0.585	0.933	0.953	0.834	0.868	1.000	0.903
55	0.208	0.631	0.944	0.948	0.821	0.887	0.998	0.867
56	0.218	0.655	0.895	0.896	0.780	0.857	0.962	0.795
57	0.218	0.643	0.828	0.783		0.817		0.713
58	0.212	0.592		0.737				
59	0.000	0.568						

Table S3: Influence of LED and detector angle setting on signal intensity of *E. coli* culture broth. V_K = 250 mL, Flask No. 6, V_L = 10 mL, n = 350 rpm, d_0 = 50 mm, T = 30°C, *E. coli* culture broth OD_{600} = 10. First row: LED angles, first column: detector angles. The signal intensity was determined by averaging the values of the horizontal signal sequence of the raw signal. The results show the relative signal intensity with respect to the highest signal intensity, from the lowest signal intensity in red to the highest signal intensity in green. Grey fields indicate angle positions for which no measurement was performed. Table adapted from Sieben (2017) (69).

[°]	124	125	126	127	128	129	130	131
51		0.635	0.911	0.933	0.878	0.918	0.958	
52	0.309	0.751	1.000	0.960	0.908	0.961	0.979	
53	0.340	0.767	0.998	0.951	0.901	0.969	0.968	0.506
54	0.352	0.786	0.972	0.925	0.873	0.948	0.932	0.451
55	0.368	0.788	0.917	0.883	0.835	0.918	0.839	0.408
56	0.367	0.766	0.883	0.826	0.764	0.857	0.738	0.324

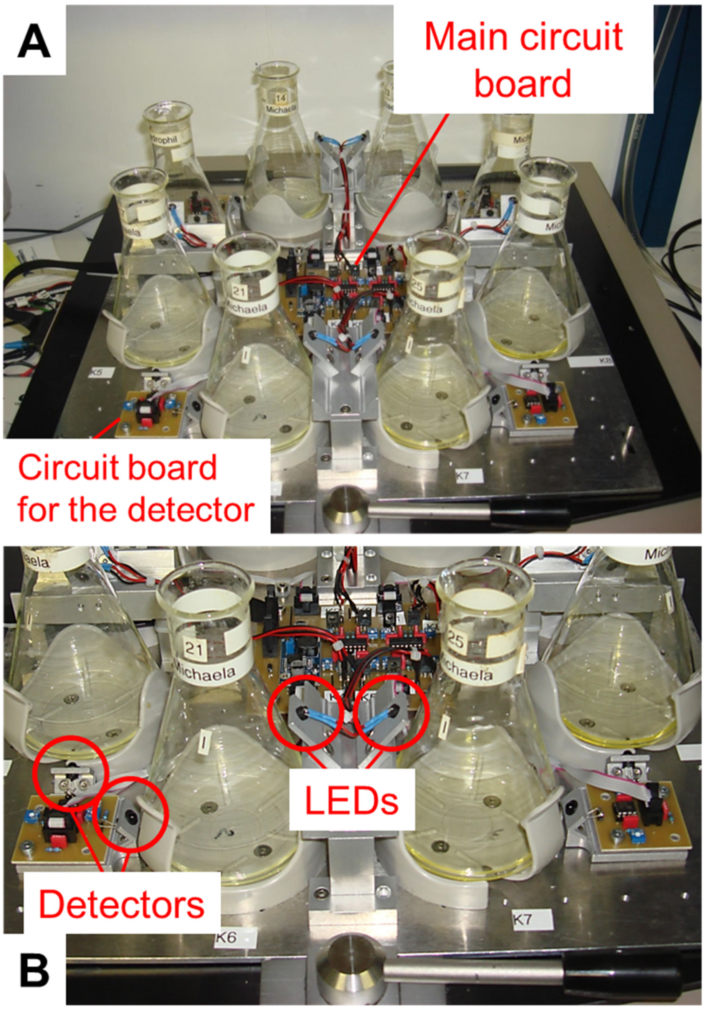

Figure S3: Transmitted light measuring set-up for eight shake flasks. (**A**) Overall view, indicating the positions of the main circuit board and the circuit board for the detector. (**B**) Close up view: Combined holders for two LEDs and two detectors, respectively. Light source (LED) and detector are on opposite sides of the shake flask. Figure adapted from Sieben (2017) (69).

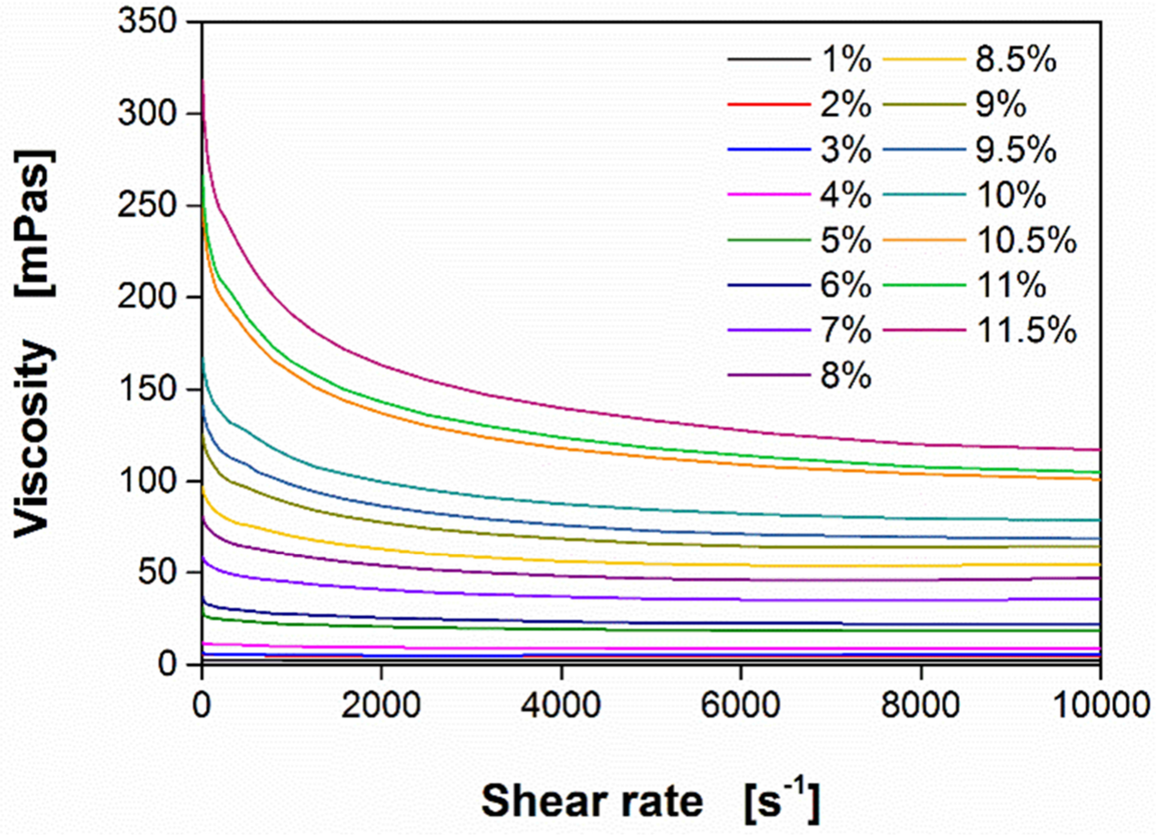

Figure S4: Shear thinning behavior of aqueous PVP solutions with different concentrations (% (w/w)). As concentration and viscosity increase, the PVP solution shows stronger shear-thinning behavior. T = 30°C. Figure adapted from Sieben (2017) (69).

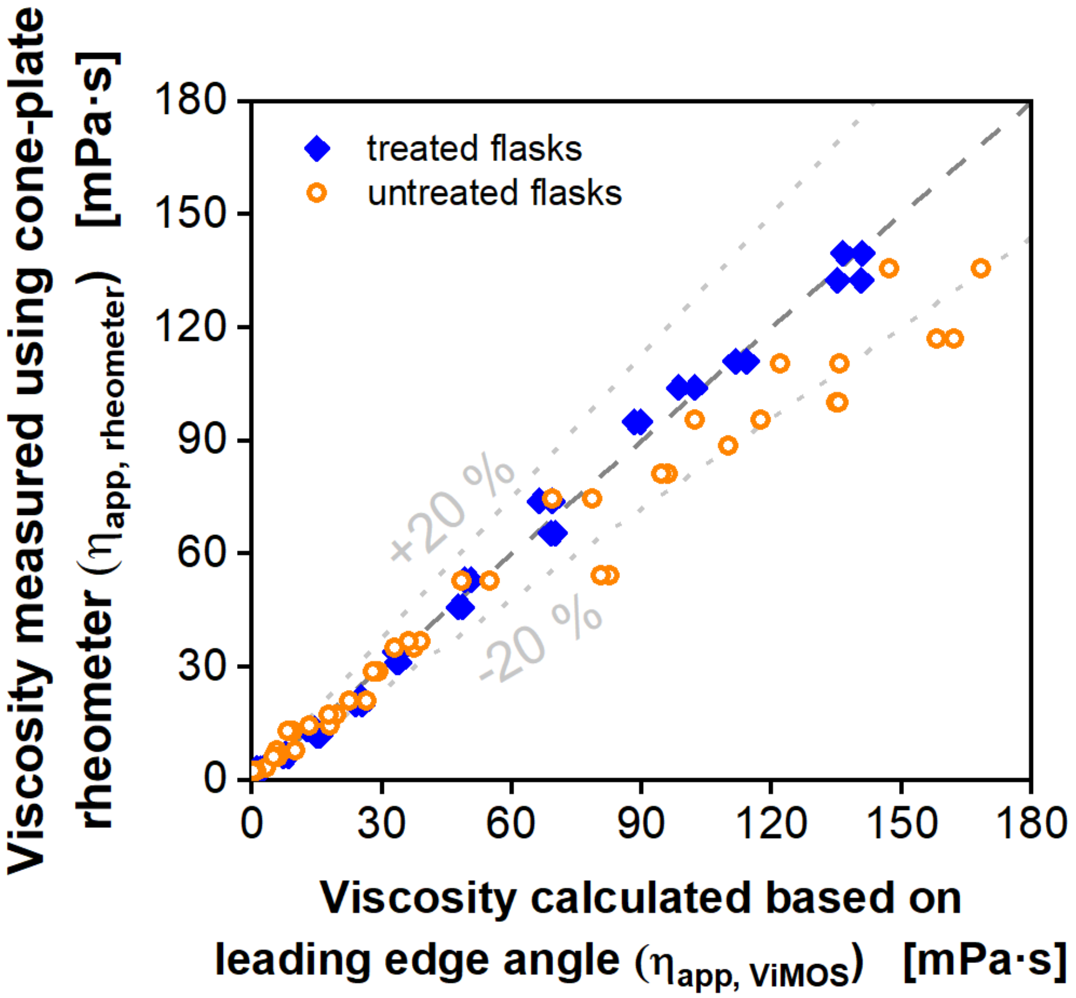

Figure S5: Parity plot of apparent viscosity calculated based on leading edge angle against measured viscosity using a cone-plate rheometer for flasks with and without nitric acid pretreatment. Influence of wetting properties of the inner flask wall on the leading edge angle of the bulk liquid θ. LED angle: 124°, detector angle: 55°. Calibration of θ with apparent viscosity based on Equation 4 using treated shake flasks for varying shaking frequencies n and filling volumes V_L. Fit parameters for calibration functions are listed in **Supplementary Table S4**.

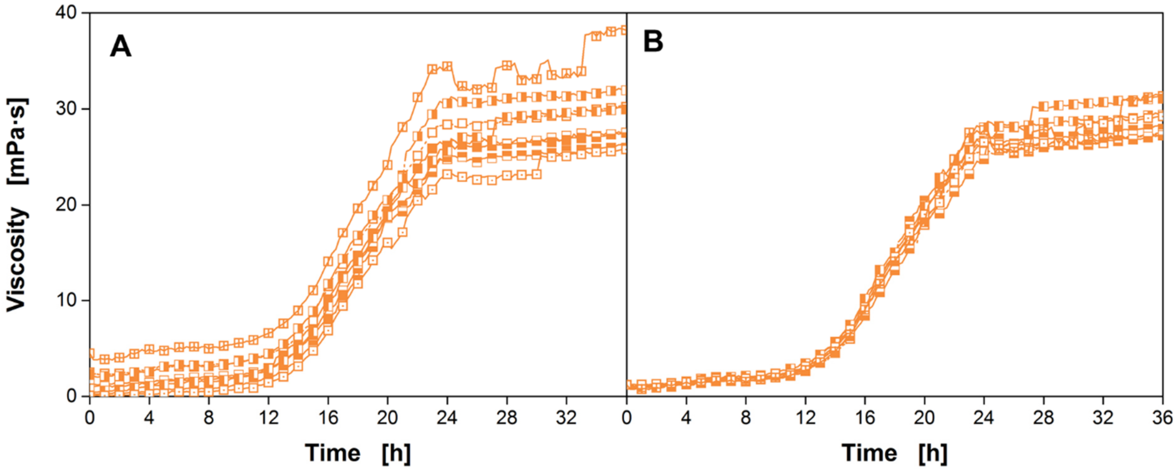

Figure S6: Correction of the impact of the shake flask wall on the viscosity determination in eight biological cultivations by zero calibration. Cultivation of *Xanthomonas campestris pv. campestris* B100, V_K = 250 mL, n = 300 rpm, d_0 = 50 mm, V_L = 20 mL, T = 0°C, GY medium. (**A**) without zero calibration (**B**) with zero calibration:

During the investigation of a suitable calibration function, it was noticed that the individual shake flasks inherently differ in their wall thickness and possibly orientation, due to their manufacturing method (**Figure 2**). These differences in wall thickness and orientation influence the online monitoring of viscous cultivations. Exemplarily, this phenomenon is illustrated by a *Xanthomonas campestris* cultivation shown in **Figure S4**. At identical conditions, the OTR and viscosity signals of the eight shake flasks in the device show exactly the same qualitative course. However, the viscosity data are vertically offset from one another. This issue can be addressed by performing a zero calibration prior to the start of the cultivation and correcting the shake flask specific offset after the cultivation. The zero calibration consists of the following steps: **1)** The viscosity of the initial sample (t = 0 h) is measured on a conventional rheometer, while the online measuring technique determines the corresponding angle θ'. **2)** The previously determined calibration function (**Equation 4**) for the selected shaking conditions containing the fit parameters a, b and c, is used to calculate the angle θ corresponding to the measured viscosity. **3)** The flask specific correction factor $\Delta\theta$ is calculated by subtracting the measured angle θ' (step 1) from the angle θ determined using the calibration function (step 2). **4)** The corrected fit parameter c' is calculated by subtracting $\Delta\theta$ from the original fit parameter c (**Equation 4**). **5)** The apparent viscosity η_{app} of the corresponding shake flask culture is calculated using the calibration function (**Equation 4**) with the fit parameters a, b and c'. Figure adapted from Sieben (2017) (69).

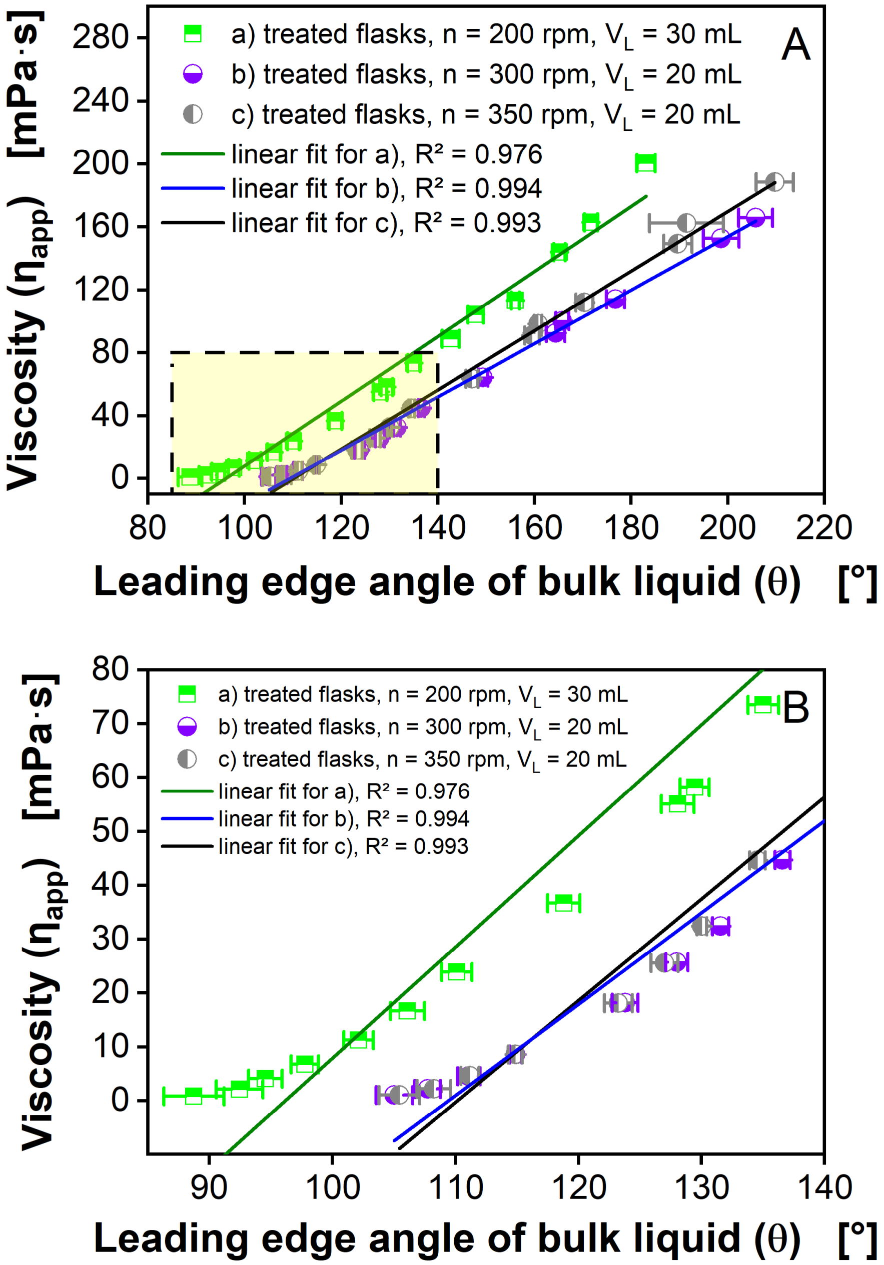

Figure S7: Linear calibration function. Calibration of θ with apparent viscosity (η_{app}) using treated shake flasks for varying shaking frequencies (n) and filling volumes (V_L). PVP solutions of different concentrations, LED angle: 126°, detector angle: 53°. V_F = 250 mL, d_0 = 50 mm, T = 30°C. Error bars represent the standard deviations of triplicates. (**B**) represents a zoomed in view of the yellow box of (**A**).

Table S4: Fit parameters for the calibration function (Equation 4) for shaking diameter d_0 = 50 mm, temperature T = 30°C and flask volume V_K = 250 mL. Table adapted from Sieben (2017) (69).

**Shaking frequency n and filling volume V_L**	**Fit parameters [-]**		
	**a**	**b**	**c**
n = 200 rpm, V_L = 30mL	2.691	0.675	87.65
n = 300 rpm, V_L = 20mL	0.756	0.946	109.93
n = 350 rpm, V_L = 20mL	0.712	0.938	110.41

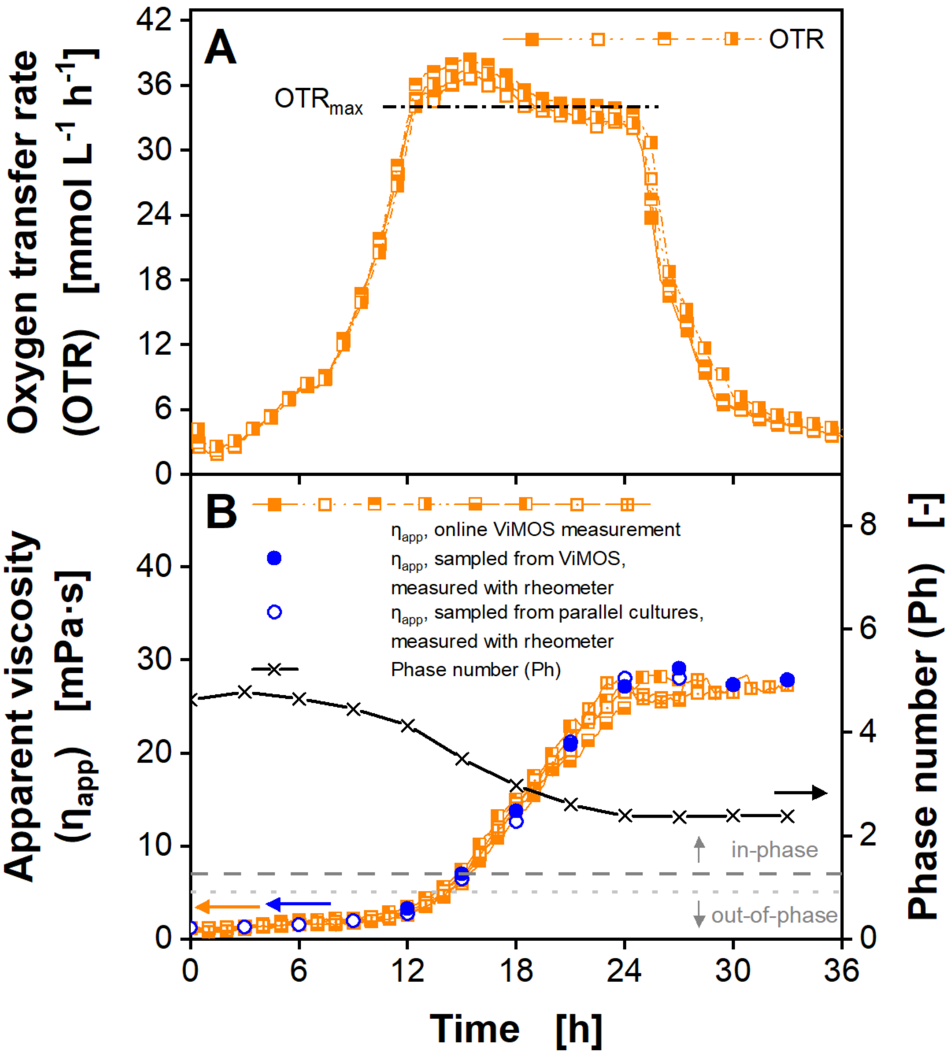

Figure S8: Cultivation of *Xanthomonas campestris pv. campestris* B100 in GY medium with simultaneous online monitoring of the oxygen transfer rate and apparent viscosity. (**A**) Oxygen transfer rates. For reasons of clarity, only every second data point is shown. Maximum oxygen transfer capacity OTR_{max} is calculated according to **Equation 3** (Meier et al., 2016) (70). Four biological replicates are shown. (**B**) Online viscosity signal and offline measured viscosity values. Every 15 min, θ was determined from 100 rotations and converted into a viscosity value by using the calibration function (**Equation 4**). Eight biological replicates are shown. For reasons of clarity, only every forth point is shown for the online signals. For offline measurements, samples were taken from both, the ViMOS and parallel normal shake flasks, incubated in a different shaker. The Phase number *Ph* is calculated based on measured viscosity according to **Equation 1** (Büchs et al. 2000) (2). The dark grey dashed line marks the critical *Ph* of 1.26 according to Büchs et al.. The light grey dotted line marks an alternative critical *Ph* according to Aizizan et al. (2019) (61). V_F = 250 mL, V_L = 20 mL, n = 300 min^{-1}, d_0 = 50 mm, T = 30°C. Parts of this figure have been adapted from Sieben (2017) (71).

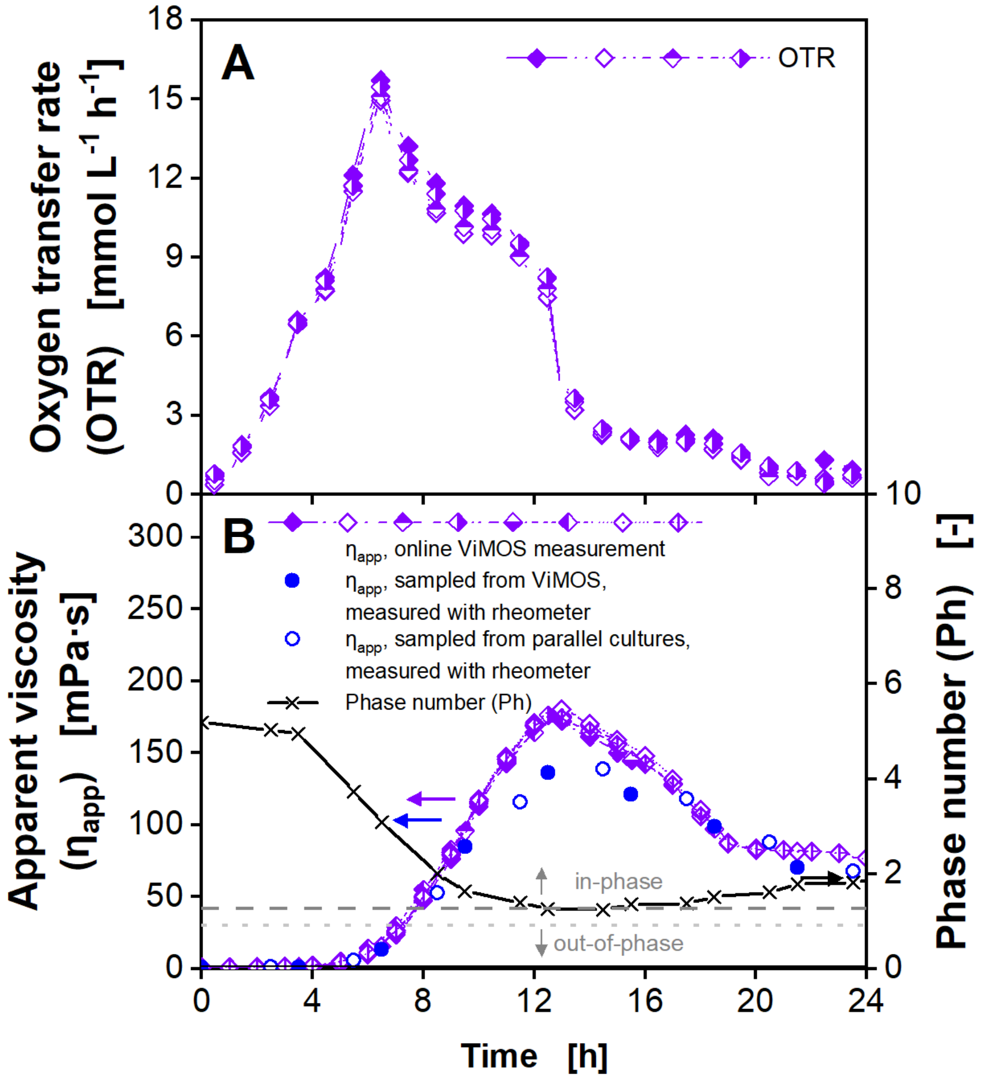

Figure S9: Cultivation of *Paenibacillus polymyxa* DSM 365 in MM1P100 medium with simultaneous online monitoring of the oxygen transfer rate and apparent viscosity. (**A**) Oxygen transfer rates. For reasons of clarity, only every second data point is shown. Four biological replicates are shown. (**B**) Online viscosity signal and offline measured viscosity values. Every 15 min, θ was determined from 100 rotations and converted into a viscosity value by using the calibration function (**Equation 4**). Eight biological replicates are shown. For reasons of clarity, only every forth data point is shown for the online signals. For offline measurements, samples were taken from both, the ViMOS and parallel normal shake flasks, incubated in a different shaker. The Phase number *Ph* is calculated based on measured viscosity according to **Equation 1** (Büchs et al. 2000) (2). The dark grey dashed line marks the critical *Ph* of 1.26 according to Büchs et al.. The light grey dotted line marks an alternative critical *Ph* according to Aizizan et al. (2019) (61). V_F = 250 mL, V_L = 30 mL, n = 200 min^{-1}, d_0= 50 mm, T = 30°C. Parts of this figure have been adapted from Sieben (2017) (71).

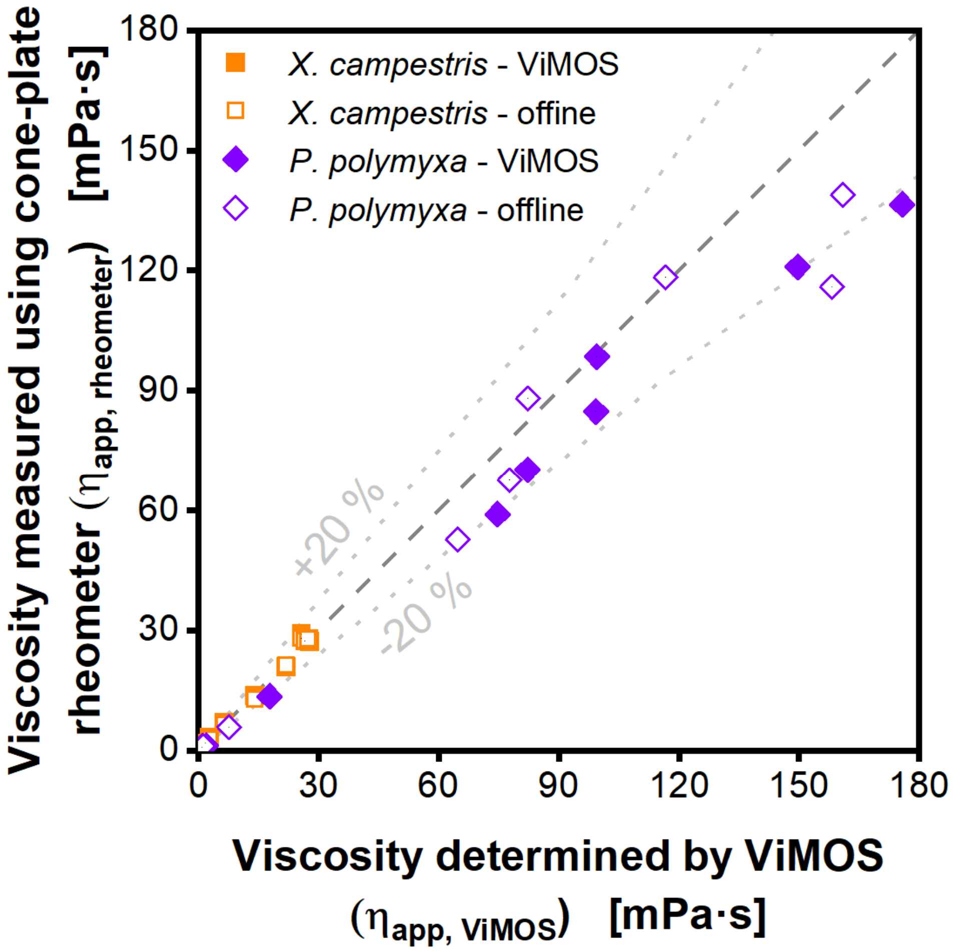

Figure S10: Parity plot of online and offline measured viscosity. Data is based on the cultivation of *Xanthomonas campestris* (**Figure 4**) and of *Paenibacillus polymyxa* (**Figure 5**). This figure has been modified from Sieben (2017) (71).

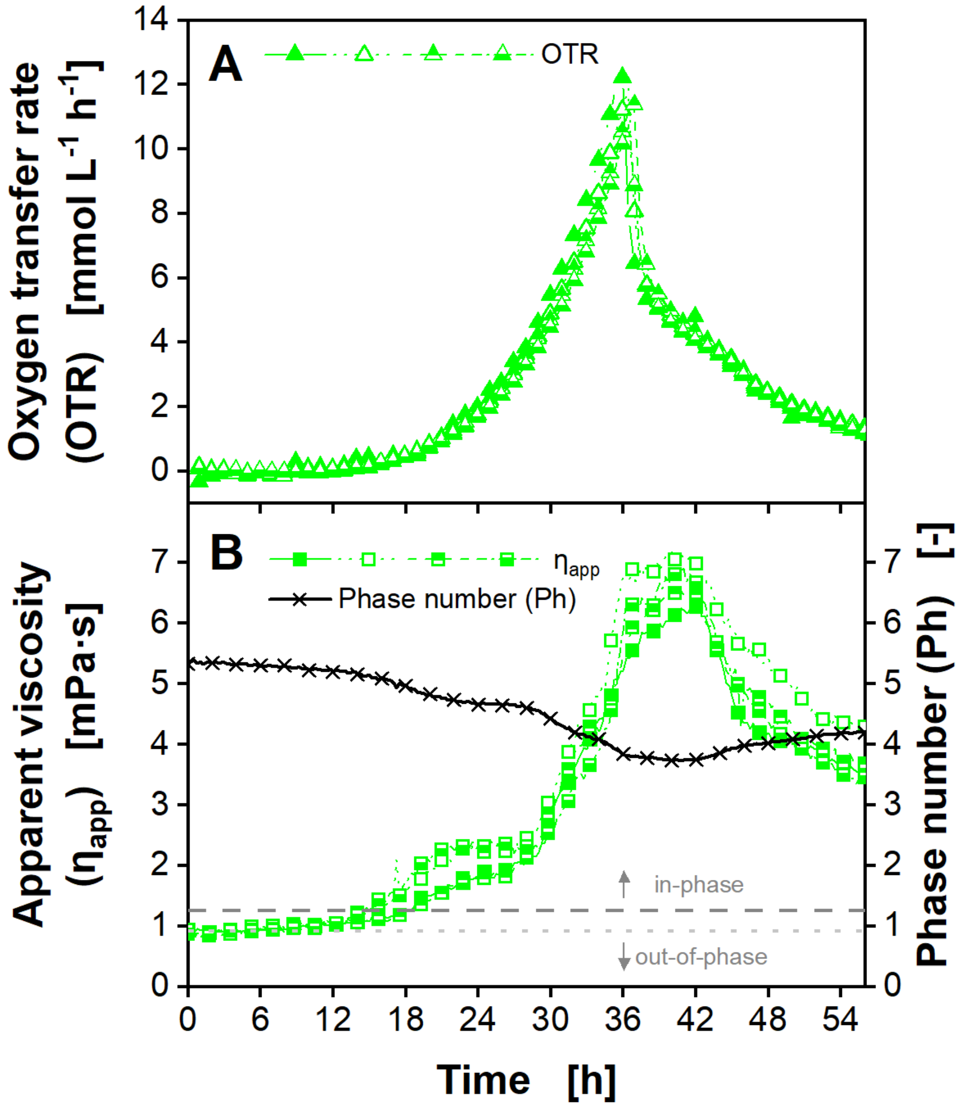

Figure S11: Cultivation of *Trichoderma reesei* RUT-C30 in modified Pakula medium with simultaneous online monitoring of the oxygen transfer rate and apparent viscosity. (**A**) Oxygen transfer rates. For reasons of clarity, only every second data point is shown. Four biological replicates are shown. (**B**) Online viscosity signal. Every 15 minutes θ was determined from 200 rotations and converted into a viscosity value by using the calibration function (**Equation 4**). Four biological replicates are shown. For reasons of clarity, only every eighth data point is shown. The Phase number *Ph* is calculated based on measured online viscosity according to **Equation 1**. For reasons of clarity, only every eighth data point is shown. The dark grey dashed line marks the critical *Ph* of 1.26 according to Büchs et al. (2000) [2]. The light grey dotted line marks an alternative critical *Ph* according to Aizizan et al. (2019) [58]. V_F = 250 mL, V_L = 20 mL, n = 350 min^{-1}, d_0 = 50 mm, T = 30°C.

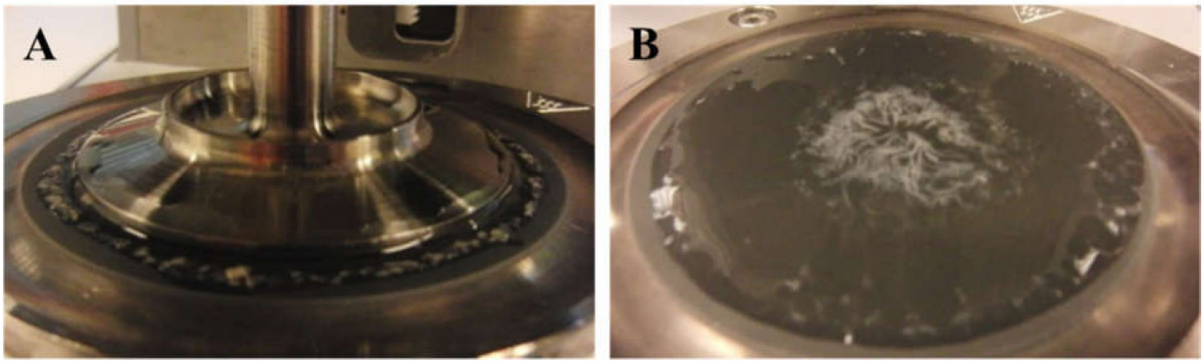

Figure S12: Rheometer measurement of *Trichoderma reesei* cultivation broth using a plate-plate rheometer setup. Fungal mycelium is destroyed by shearing between the two plates. T = 30°C, gap size = 0.1 mm. (**A**) During measurement, (**B**) after measurement. Figure adapted from Sieben (2017) (69).

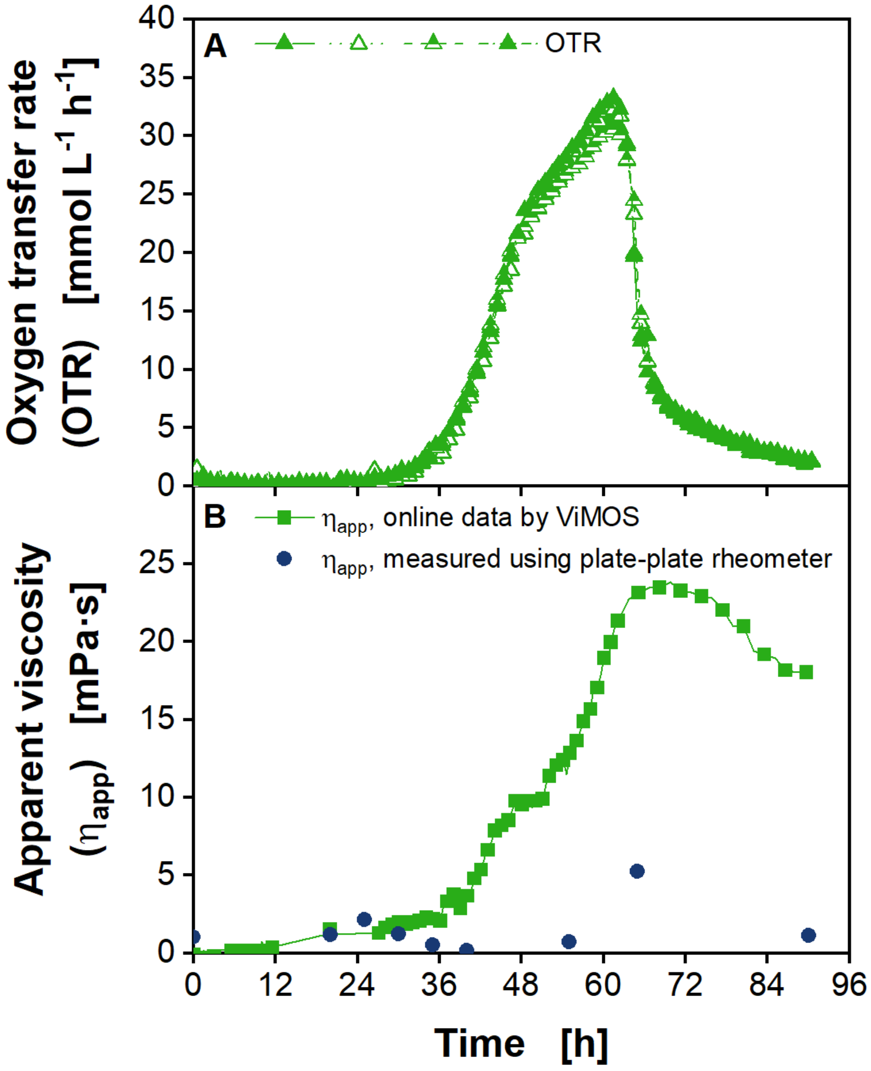

Figure S13. Cultivation of *Trichoderma reesei* RUT-C30 with simultaneous online monitoring of viscosity and offline sampling. V_K = 250 mL, n = 350 rpm, d_0 = 50 mm, V_L = 20 mL, T = 30°C, modified Pakula medium with 50 g/L glucose and 100 mM PIPPS buffer. For comparison, the *T. reesei* cultivation shown in **Figure 6** was performed using 30 g/l glucose and 100 mM MES buffer. (**A**) Oxygen transfer rates. For reasons of clarity, only every second data point is shown. (**B**) Offline measured viscosity values and online viscosity signal determined by the single shake flask measuring set-up (see **Figure S1**). Every 30 minutes, the average of the leading edge angle was determined from 100 rotations and converted into a viscosity value by using the calibration function (**Equation 4**). For reasons of clarity, only every second data point is shown for the online signals. LED angle was set to 125°, the detector angle to 55°. Between 12 and 25 hours, data points are missing due to software failure. Offline samples were measured using a plate-plate rheometer. Figure adapted from Sieben (2017) (69).
